# Virtual Reality and Empathy Enhancement: Ethical Aspects

**DOI:** 10.3389/frobt.2020.506984

**Published:** 2020-11-09

**Authors:** Jon Rueda, Francisco Lara

**Affiliations:** FiloLab Scientific Unit of Excellence, Department of Philosophy, University of Granada, Granada, Spain

**Keywords:** virtual reality, empathy, empathy enhancement, moral enhancement, moral psychology, neuroethics, applied ethics, virtual embodiment

## Abstract

The history of humankind is full of examples that indicate a constant desire to make human beings more moral. Nowadays, technological breakthroughs might have a significant impact on our moral character and abilities. This is the case of Virtual Reality (VR) technologies. The aim of this paper is to consider the ethical aspects of the use of VR in enhancing empathy. First, we will offer an introduction to VR, explaining its fundamental features, devices and concepts. Then, we will approach the characterization of VR as an “empathy machine,” showing why this medium has aroused so much interest and why, nevertheless, we do not believe it is the ideal way to enhance empathy. As an alternative, we will consider fostering empathy-related abilities through virtual embodiment in avatars. In the conclusion, however, we will examine some of the serious concerns related to the ethical relevance of empathy and will defend the philosophical case for a reason-guided empathy, also suggesting specific guidelines for possible future developments of empathy enhancement projects through VR embodied experiences.

## Introduction

From the very beginning, the contemporary debate on moral enhancement has predominantly been a discussion about moral bio-enhancement, that is, the ethical considerations of using biotechnologies such as gene editing, pharmacological drugs, or neurological interventions in order to improve our moral behavior (Douglas, 2008, 2013; Faust, 2008; Persson and Savulescu, 2008, 2012; DeGrazia, 2014; Harris, 2016). More recently, moral enhancement through the use of Artificial Intelligence is increasingly being considered (Whitby, 2011; Savulescu and Maslen, 2015; Klincewizc, 2016; Giubilini and Savulescu, 2017; Lara and Deckers, 2020). One of the aims of this research paper consists of broadening the horizons of the moral enhancement debate. We will address the use of Virtual Reality (VR) for that purpose—an increasingly popular and widespread form of technology that may have a powerful impact on human morality.

Although several ethical issues raised by this technology have already been widely debated in recent years^1^, we will focus on its use for moral enhancement, particularly on how VR could improve empathy. Empathy has been one of the recurrent objectives of traditional moral education. We will show that VR is a technology that overcomes some of the limitations of conventional methods that attempt to enhance empathy. However, empathy is an important psychological ability that is not beyond the scope of ethical controversies. The fact that VR is a strong candidate for promoting empathy makes the ethical scrutiny regarding its potential promises and drawbacks in the domain of human moral enhancement even more important^2^.

Our argument rests as follows. First, we will present an overview of what VR is, the devices that make it work and the essential concepts needed to properly understand it. In the Virtual Reality as an “Empathy Machine” section, we will offer a definition of empathy and present its main subtypes. After that, we will examine the concept of VR as an “empathy machine,” a notion which has aroused a great deal of enthusiasm regarding the potential of this medium in a variety sectors. In our opinion, despite the fervor unleashed in civil society by this technology, most of the social activist projects pursuing this concept of VR (beyond raising awareness of particular societal problems) do not represent the most promising method for enhancing empathy itself. This is partly because there is little empirical evidence supporting their impact on altruistic or prosocial motivations and because of ethical caveats regarding the predominantly emotional content of these audiovisual experiences. In order to overcome these previous examples, in the Virtual Reality Embodied Perspective-Taking, we will review an emerging experimental body of literature that is studying the use of embodiment in different VR avatars in order to improve mostly empathy-related phenomena. In principle, this could be a promising way of enhancing empathy through VR because there is evidence of their potential to reduce biases and discriminatory attitudes and to promote morally altruistic initiatives. In the Further Ethical Aspects: in Defense of a Reason-Guided Empathy, we will deal with the ethical concerns that arise in connection with the goal of fostering empathy. We will present some of the recurrent doubts regarding the moral significance of empathy and propose some requirements to counter these alleged shortcomings. Above all, we will advocate a reason-guided empathy for the development of the future attempts to enhance empathy through embodiment in VR experiences.

## Virtual Reality Technologies: Devices and Fundamental Aspects

Virtual Reality^3^ has historically been referred to as a set of technologies providing immersive experiences in computer-generated worlds. The most important features of computer-based VR are, on the one hand, the interactivity with digital scenarios, objects and avatars and, on the other, the motion tracking that permits the rendering of movement in a virtual environment from a first-person perspective. Today, the increasing popularity of the display of 360° videos in VR headsets has sparked debate regarding whether or not this practice should also be considered VR. 360° VR films—or so-called “cinematic VR” (Archer and Finger, 2018)—do not have these features, but they do still offer immersive experiences. As in computer-generated virtual environments, participants can look in any direction inside an immersive 360° video by moving their head in a natural manner, that is, supporting sensorimotor contingencies that follow the principles of how we perceive the real world (Slater, 2009). However, a computer-generated world is technically more immersive than a 360° VR video in the sense that the latter could be simulated by the former, but not vice versa (Slater and Sanchez-Vives, 2016, p. 35–37; see also Slater, 2009, p. 3350–3351). This also means that both technologies can serve different purposes; for instance, cinematic VR has great potential for storytelling. 360° VR videos sometimes have interactivity possibilities which, while not capable of affecting the environment, do contain features such as the ability to select different courses of action to alter the storylines. Moreover, 360° video-based VR depictions are, by default, photorealistic compared to computer-generated graphics. In this article, we will consider VR in a broad sense that accommodates a plurality of immersive experiences created by different types of VR technology.

The most famous apparatus in the development of VR is the *Head Mounted Display* (HMD). HMDs are helmets, goggles, or headpieces that display images of a digitally rendered world for each eye, creating a fully immersive experience with a three-dimensional stereoscopic view. HMDs also provide the display of 360° VR videos and are the most popular VR devices due to the commercial success they have achieved in recent years. In a VR multisensory experience, HMDs provide the visual element. Sound is often transmitted by means of headphones/earphones or an external speaker, thus helping to increase the degree of immersion. Touch or vibrotactile signals are also part of some VR experiences through haptic feedback devices. There are some gadgets (handheld controllers, force-feedback joysticks, data gloves, datasuits, etc.) which are essential to the manipulation of virtual objects as well as for the sense of touch. Some VR labs and room-scale VR, such as the Virtual Human Interaction Lab led by Jeremy Bailenson at Stanford University, include a vibrating floor and can, for instance, simulate earthquakes (Bailenson, 2018). In summary, sight, sound, and touch are the most common sensory base of VR experiences. The stimulation of olfactory and gustatory senses in VR is still a subject of study rather than a current possibility. Nevertheless, its future existence cannot be discarded (Slater and Sanchez-Vives, 2016; Oh and Bailenson, 2017).

Along with HMDs, there are other devices that enable the vivid illusions that VR provides. For instance, there are a variety of devices related to motion tracking which are fundamental to the interaction with virtual environments and to the position and orientation of the subject. These apparatuses provide real-time information to the computer in order to render the virtual world in accordance with the physical movements of the protagonist of the experience and “to update the rendered first-person perspective viewpoint accordingly” (Ahn et al., 2016, p. 6). Although HMDs are in charge of the *display*, VR would not be possible without the *tracking* and *rendering* permitted by other hardware. As Oh and Bailenson (2017, p. 2) note: “[t]here are different types of tracking systems: marker-based systems (e.g., with mechanical, magnetic, optical, inertial trackers), computer vision systems (e.g., Microsoft Kinect), and data gloves.” Body-tracking suits are also noteworthy in this aspect (Peck et al., 2013, p. 780).

In this article, we are particularly interested in highlighting the importance of HMDs because the expression “empathy machine,” as well as the experiments that we will address in the Virtual Reality Embodied Perspective-Taking, are based on this VR technology. Still, it should not be forgotten that there are other types of VR systems which are not HMDs but are nevertheless important in the historical development of VR and are still being used in some labs as well as in commercial applications. There are various technologies that permit navigation, exploration, and interaction in virtual scenarios. For instance, the *Cave Automatic Virtual Environment* (CAVE) is a virtual, immersive and interactive space (Cruz-Neira et al., 1993). The CAVE system is based on a cube-shaped room where videos and virtual representations are projected on the walls. A special type of glasses allow for the visualization of objects in three dimensions. In fact, CAVEs “became one of the mainstays of VR research and applications from the late 1990s and through the 2000s until recently” (Slater and Sanchez-Vives, 2016, p. 4). Furthermore, the use of *desktop VR*, based on two-dimensional computer screens, is also worth noting (Parsons, 2015, p. 2). These examples are less immersive than HMDs on a “scale of immersiveness,” as Slater and Sanchez-Vives (2016) point out, because an HMD could simulate a virtual experience of a CAVE or a desktop VR, but not vice versa. Recently, specific types of *cardboard* have also been used as a rudimentary but economical way to provide virtual experiences. Cardboards are easy to assemble and low-cost headset platforms that work with smartphones and mobile apps, providing a 3D stereoscopic view. Moreover, market forces are driving the innovation of updates that aim to improve the VR experiences of home users at more affordable prices. There are now all-in-one headsets available (e.g., Oculus Go or Quest) that do not require VR-enabled PCs or smartphones and which offer high-quality immersive experiences more accessible to a broader audience. With these examples, far from an exhaustive list, we hope to have provided a general conception of the rich field of VR technologies.

Based on the above review of the main devices usually included under the category of VR, we will now introduce the main characteristics that allow us to unify them under such a denomination and to distinguish them from one another according to the degree to which they possess these differentiating properties.

One important characteristic of VR is *immersion*. Immersion refers to “the technical capability of the system to deliver a surrounding and convincing environment with which the participant can interact” (Sánchez-Vives and Slater, 2005, p. 333). Immersion is a technological quality that may differ from one type of VR system to another. It is dependent on the technological affordances of each VR system (Slater and Sanchez-Vives, 2016). Some VR systems have characteristics (e.g., higher display resolution, more extensive tracking) that qualify them as more immersive than others. Thus, there are different degrees of immersion that can be measured in an objective manner, regardless of the human experience that it provokes (Sánchez-Vives and Slater, 2005; Oh and Bailenson, 2017; Herrera et al., 2018). It is measurable because immersion refers to the technological quality of the media of a particular VR apparatus in relation to its capacity to remove us from the physical reality^4^ and therefore elicit different levels of presence. According to Cummings and Bailenson (2016), immersion can be measured by taking into account different variables, such as tracking level, stereoscopic vision, image quality, field of view, sound quality, user perspective, resolution, etc.

Immersive systems and their particular technological affordances also support a set of valid actions known as *sensorimotor contingencies* (Slater, 2009). For instance, participants using immersive headsets such as HMDs rapidly learn the effects of head movement in gaze direction because the head tracking system renders the visual perception according to the real movements of the user. However, imagine that the participant reaches to grasp a virtual object; in the absence of a haptic system or data glove, it will be impossible to feel anything because the manipulation of objects and the sense of touch are not valid sensorimotor contingencies in that VR experience. That said, the technological affordances of immersive VR can create realistic illusions and sensorimotor contingencies that other technology-based mediums such as desktops or TVs cannot simulate to the same degree. This is because theoretically, in principle, it is possible to create Immersive Virtual Environments (IVEs) in which to “fully simulate what it is like to go into a non-immersive system” (Slater, 2009, p. 3350). Moreover, a commonly held belief is that IVEs offer multisensory experiences, free navigation, and interactive possibilities in three-dimensional and digitally-rendered worlds from a first-person perspective, as if we were in the physical reality, but without being exactly in the physical reality (Bailey et al., 2014, p. 573; Ahn et al., 2016, p. 2; Oh et al., 2016, p. 400; Herrera et al., 2018, p. 4). This contraposition between the virtual world and the physical or non-virtual world is a core characteristic of immersion.

Another characteristic of VR is *presence*. This refers to the psychological experience of “being there” (Heeter, 1992). In virtual environments, presence is understood as place illusion—the qualia of being located inside the virtual word (Slater, 2009). In contrast to immersion, presence is considered a subjective factor (Sánchez-Vives and Slater, 2005; Oh and Bailenson, 2017). If immersion is the technical capability of the VR technology, presence is the psychological response to it (Sánchez-Vives and Slater, 2005). Consequently, the sense of presence is conditioned by the degree of immersion that each VR technology can offer. For instance, technological affordances such as tracking level, wider fields of view of the display and the use of stereoscopic visuals are generally more impactful with regard to the sense of presence than the quality of visual images or auditory content (Cummings and Bailenson, 2016). Furthermore, presence is related to the extent to which VR users feel that they are in a specific environment (Shriram et al., 2017, p. 312). One interesting point is that, when the feeling of presence is well-achieved, IVE participants react, behave and feel as if they were in non-virtual situations (Slater et al., 2006; Felnhofer et al., 2015, p. 49; Oh and Bailenson, 2017, p. 6–7). Our motor, perceptual and physiological systems function in virtual scenarios in the same way that they do in the “real world” (Bailenson, 2018, p. 19–20). The subjective illusion of presence, the sensation of “being there,” is one of the psychological features that makes VR work, “*in spite of the fact that you know for sure that you are not actually there*” (Slater and Sanchez-Vives, 2016, p. 5, italics in the original source). It is therefore not an exaggeration to claim that “presence is the sine qua non of VR” (Bailenson, 2018, p. 19).

In addition, there is another subjective experience that VR can create, through the use of HMDs, which is the sense of *embodiment*. Embodiment is the sense of experiencing the body (or some body parts) as “one's own” —such that this type of bodily illusion is also frequently referred as “body ownership.” The feeling of body ownership can be achieved by inhabiting other avatars. Avatars are digital representations that participants of VR experiences can control with their actions (Won et al., 2015b, p. 6). The body transfer to an avatar consists of replacing our physical body with a virtual one. One of the building blocks to obtaining a “full body ownership illusion” is the first-person perspective (Maselli and Slater, 2013), since visual confirmation of the body substitution is one of the grounds for the virtual body transfer (Slater et al., 2010; Banakou et al., 2016, p. 7; Slater and Sanchez-Vives, 2016, p. 8). Another underlying mechanism of the sense of embodiment is congruent body tracking (Maselli and Slater, 2013). The tracking and rendering systems allow the VR headset to display the synchronized movements of our “real bodies” and the virtual ones. In other words, an accurate tracking of the congruent visual motor correlations between the “real body” and the fake virtual body are important to induce embodiment. For instance, the use of mirrors in virtual experiences is very common in order to familiarize participants with their novel virtual appearance and to confirm that the virtual body moves synchronously with the physical one. Another mechanism that induces the sense of embodiment is agency; that is, experiencing the ability to control the body in order to act in the virtual environment leads to stronger body ownership illusions (Seinfeld et al., 2020). Most importantly, it could be said that a realistic sense of body ownership has been achieved when the body transfer causes “the brain to generate the illusion that the virtual body is one's own” (Slater et al., 2010, p. 6), or, what is almost the same, that the “person has become the virtual body” (Ahn et al., 2016, p. 4).

This fact is very important because several studies have provided supporting evidence that changing the virtual body can lead to changes in self-conception (see Maister et al., 2015). The process of self-identification with the virtual avatar alters social cognition and behavior in IVEs and, as we will see with some virtual embodiment experiments in the Virtual Reality Embodied Perspective-Taking, some changes can even be detected in the implicit attitudes in the “real world” following a virtual experience. Yee and Bailenson (2007) coined the term “proteus effect” to explain these phenomena of digital self-transformation. The proteus effect refers to the psychological phenomenon of how changing our self-identity for another social representation can accordingly change our behavior because the subject ascribes the social stereotypes and beliefs linked to the novel identity adopted. Yee and Bailenson (2007) studied, for instance, how embodying an attractive virtual avatar leads to more confident behavior and, from proxemics point of view, stepping closer into the personal space of the virtual interlocutor. Although it was originally conceived to explain the behavior-altering effects of using virtual avatars in computer games, there is evidence that the proteus effect also applies to fully embodied VR experiences (see Farmer and Maister, 2017). If an avatar is the primary identity of the virtual participant (Yee and Bailenson, 2007), the user might assume some of the semantic properties, implicit attitudes and behaviors that are often represented by that social identity when the sense of embodiment occurs in an avatar with out-group visual appearance (Seinfeld et al., 2020). Similarly, with regard to the potential of virtual embodiment to significantly alter social cognition and behavior, in the Virtual Reality Embodied Perspective-Taking we will address a series of experiments that show the potential of VR for promoting empathic perspective-taking or altruistic motivations through the embodiment of participants in other virtual avatars.

Moreover, body transfer might be provoked even if the virtual avatar does not have a human appearance. Jaron Lanier invented the expression “homuncular flexibility” to refer to the high malleability of our body schema in VR experiences (Won et al., 2015a,b). We have the mental plasticity to adopt different virtual body representations that are not necessarily human, or that look like humans but have, for instance, a third arm, or even stranger experiences such as having eight limbs like a lobster (Won et al., 2015b). However, human-like bodily appearance is one of the building blocks of full body ownership illusion (Maselli and Slater, 2013), meaning that the sense of embodiment may decrease in non-human avatars compared to realistic virtual human bodies (see also Seinfeld et al., 2018).

Finally, the last point of this characterization of VR is related to this same idea of “unnatural” experiences. VR is an experience generator (Bailenson, 2018, p. 46) and a simulation medium (Ramirez and LaBarge, 2018). It offers the possibility of simulating events that may be experienced in physical reality. VR is, in this way, a simulation medium because it can mimic experiences that have their equivalent in the “real world.” However, VR is not confined to the reproduction of “real” events, objects or experiences. The potentials and perils of VR are enormous because it can be used to simulate experiences that are part of our common reality, situations that we are unlikely to experience in our lives or even situations that are simply impossible to experience in physical reality (Slater and Sanchez-Vives, 2016). The possible experiences that may be achieved in VR are therefore almost infinite (Oh and Bailenson, 2017, p. 7). What *could* be experienced in VR is subject to human imagination, VR designers and content developers, but what *should* be experienced in VR is an issue that must be discussed from an ethical perspective. In the next two sections we will examine how VR could be used to intentionally promote empathy, addressing the ethical considerations that arise as a consequence of this goal.

## Virtual Reality as an “Empathy Machine”

In this Virtual Reality as an “Empathy Machine” section we will approach the characterization of VR as an empathy machine. First, we will define empathy and introduce its main subtypes. Then, we will explain the origin of the tag “empathy machine,” offering some examples of visual artists, storytellers and organizations that have enthusiastically embraced this conception of VR. VR is, in this sense, an appealing communication medium for those wishing to offer powerful narratives or captivating experiences, opening new perspectives to address social and environmental problems. Nevertheless, we will argue that, from an ethical standpoint, this is *not* the most reliable way to enhance empathy.

*Empathy* is an ability that encompasses diverse psychological processes related to sharing and understanding the internal (mainly affective) mental states of other (not only human) beings. Nonetheless, it is not a one-dimensional phenomenon. The different uses assigned to the term “empathy” often elicit non-equivalent meanings (Batson, 2009; Cuff et al., 2016). Thus, according to the widely used model formulated by Davis (1980), we can distinguish, in principle, four differentiable types of empathy: fantasy (necessary to identify with fictitious characters), empathic concern, personal distress, and perspective-taking. However, in the literature regarding VR and empathy, there has been a general tendency to focus on the two latter types due to the simpler distinction between emotional empathy (personal distress) and cognitive empathy (perspective-taking) (Fisher, 2017, p. 236–237; Ramirez, 2017, p. 510–511; Hamilton-Giachritsis et al., 2018, p. 2; Francis et al., 2018, p. 5; Seinfeld et al., 2018, p. 1; van Loon et al., 2018, p. 2; Bailenson, 2018, p. 79–80; Schoeller et al., 2019, p. 1)^5^.

On the one hand, personal distress would be the emotional empathy par excellence. It is a reflexive and spontaneous disposition that leads to the mirroring or contagion of other people's negative affective states (emotions such as suffering, sorrow, etc.). It allows one to share the feelings of another, together with a clear and simultaneous self-other distinction (Bertrand et al., 2018; Schoeller et al., 2019). By “share” we do not mean experiencing exactly the same feeling, but having at least an “image” or a “sensuous representation” of what the other person is feeling (Persson and Savulescu, 2018, p. 186). In many cases, this disposition of contagion is facilitated by an instinctive imitation of bodily postures (Chartrand and Bargh, 1999), facial expressions (Ekman, 1992; Ekman and Davidson, 1993), and emotional tone when talking to others (Neumann and Strack, 2000). The emotions aroused by the contagion entail a personal distress which, in a desperate and self-interested attempt to evade it, can lead to involvement in order to alleviate the pain of the other (Batson, 1991; Batson and Oleson, 1991).

On the other hand, perspective-taking is understood as the active effort to put oneself in another person's shoes, thinking about the causes of the other person's affective states, or just trying to imagine how one can adopt the other person's beliefs and states. This is a less spontaneous and less “selfish” psychological disposition. As such, the observer reacts based on what the other says and does, as well as his or her own knowledge of the person's character, values and desires (Darwall, 1998). It is therefore an essentially cognitive form of empathy.

Actually, (Bailenson, 2018, p. 79–80; following Zaki, 2014) also refers to a third type of empathy, which is mainly motivational. It is similar to the one that Davis (1980) called “empathic concern.” Davis defined it as the tendency to experience feelings of warmth, compassion and concern for others undergoing negative experiences. In the case of the person showing empathic concern, there is therefore an attempt to adopt the perspective of the other who is motivated by an interest in his or her well-being. Here, the predominant element is not the personal point of view of the observer but rather the impersonal point of view from which one matters as much as the other (Darwall, 1998, p. 263). The aim is to feel on the behalf of the other (Zahn-Waxler and Robinson, 1995). Precisely because of this, this type of empathy is the core source of altruistic motivation in Batson's (1991), 2015) “empathy-altruism hypothesis.” This third motivational type of empathy, while primarily emotional, can be elicited by either of the aforementioned types. Moreover, we should bear in mind that empathy “in its fullest form includes both the cognitive and affective dimensions” (Simmons, 2014, p. 98). These dimensions are sometimes difficult to delineate plainly and there are cases in which they can overlap. For instance, a cognitive exercise of perspective-taking might lead to a better recognition of the emotions of other beings^6^, with the subsequent motivation due to the concern for their suffering. Therefore, although it is important to differentiate these types of empathy, it should not be neglected that they are not only intertwined but also frequently co-present (Read, 2019).

Now, the cliché spread by the idea that VR is an *empathy machine* has not been popularized from the academic sphere. Chris Milk, a filmmaker, storyteller and visual artist, made a vigorous claim when he deemed VR “the ultimate empathy machine” in a 2015 TED talk that went viral (Milk, 2015). Together with Gabo Arora, he is the cocreator of *Clouds Over Sidra* (Arora and Pousman, 2015) a short film sponsored by the United Nations, in 360° VR video format, which tells the story of Sidra, a 12-year-old Syrian girl in a refugee camp in Jordan. It aims to raise awareness of the humanitarian crisis provoked by the war in Syria. The United Nations High Commissioner for Refugees (UNHCR) has also used VR in several funding and awareness campaigns^7^. In addition, other civil society organizations have pursued the idea of VR as an empathy machine. For instance, international animal rights NGOs such as Animal Equality (which has set up the project *iAnimal* using VR to transport people to real slaughterhouses and intensive breeding farms to witness the life of many animals in the meat and dairy industry from a first person perspective^8^) and People for the Ethical Treatment of Animals (with the VR projects *I, Chicken, I, Orca*, and *Eye to Eye*) illustrate the power of VR to foster empathy, in those cases, for non-human beings. Some large companies have also launched various projects focusing on the potential of VR for social transformation such as HTC's *VR for Impact* or Oculus's *VR for Good*^9^^,^^10^.

The interpretation of VR as the *ultimate* empathy machine may seem exaggerated, but there is an element of truth to it. For instance, Herrera et al. (2018, p. 2) acknowledge the conception of VR as an empathy machine “since it allows people to viscerally experience anything from another person's point of view.” In addition, Bollmer (2017, p. 63) considers that the term “empathy machine” is very often used to characterize VR because it “refers to any attempt to make sensible to oneself the emotional experience of another via technology, often with the goal of inhabiting another body.” VR could be used, therefore, as a technological means of triggering very different empathic processes in participants.

It is worth mentioning that many of the previous examples of the uses of VR as an empathy machine stand out for their impact on emotional responses and the consequent objective of fostering emotional empathy (personal distress) and/or empathic concern. We have already noted in the Introduction section that 360° VR videos are a promising tool for storytelling. In this sense, many advocates of the empathy machine concept have viewed VR as a particularly effective medium in communicating emotionally-charged stories. Many of the 360° VR videos that are driven by mass-consumption and which seek to provoke empathy in specific targets, raise awareness of specific social problems, or change mindsets in relation to certain issues, are also compelling narratives that elicit highly emotional reactions in users. Following Fisher (2017, p. 233–236), VR often produces so-called “empathic actualities,” that is, emotionally-charged experiences whose impact could sometimes result in sympathy or compassion^11^.

In our opinion, we should be wary of these VR empathy machine projects for two reasons. The first is that we still have little empirical evidence on the effects of these VR cinematic experiences and, moreover, from the little we do have, there seems to be little difference in terms of empathy between 360° VR and watching the same video on a screen. The second reason for limiting expectations is that there are certain ethical caveats linked to the emotional component of these experiences.

With regard to the first, some studies deflate the hype of 360° VR as the *ultimate* empathy machine. Bang and Yildirim (2018) showed that experiencing the documentary *After Solitary* in a commercial VR headset was not substantially different from seeing it on a desktop computer via YouTube 360° in terms of the resulting state empathy levels. In Archer and Finger (2018), both immersive conditions and non-immersive conditions outperformed the text condition in eliciting empathic responses. However, there was no statistically significant difference between the two; that is, displaying the video on an HMD was not much better in producing empathy than on a two-dimensional desktop monitor. Similarly, with regard to immersive journalism, Sundar et al. (2017) compared three storytelling media showing that participants in VR and non-immersive 360° video conditions were more empathic toward the characters than when reading the text on which the stories were based (the New York Times' *The Displaced* and *The Click Effect*), there being an unsubstantial difference between VR and 360° video. Weinel et al. (2018) also found in a study on autism that VR cardboards offer limited benefits over 360° YouTube video formats with regard to generating empathy. An exception to these results is the study of Schutte and Stilinović (2017) in which the VR format resulted in greater engagement and a higher level of empathy (empathic perspective-taking and empathic concern) for the refugee protagonist of the previously mentioned *Clouds Over Sidra* than a control two-dimensional format. Overall, most of the previous studies show that, while cinematic VR can certainly promote empathy, the differences between this medium and the desktop video format are not as vast as some theorists of the VR as an empathy machine might dream. Still, these studies have some limitations. Almost all have been conducted with university students or young participants, the size of the samples is predominantly small—except for Archer and Finger (n: 180) and Sundar et al. (n: 129)—and the moderating effects of first-exposure and familiarity with VR were largely underexplored—again except for Archer and Finger, who suggested that novelty or over-familiarity with the VR experience, as well as choice of topic, correlated with the level of empathic responses. Consequently, one thing remains clear: further empirical evidence is needed to validate the distinctive potential of cinematic VR over other media to prompt empathic responses. To this end, in the next section, we will present some virtual embodiment experiments that provide more promising empirical support of empathy-related altruistic and prosocial motivations after the virtual experiences.

With respect to the second, the fact that 360° VR might have a stronger impact on emotions than other mediums is important from an ethical point of view. If the content is highly tendentious, modifying the emotions of the user directly (without providing contrasted information or serious data about reality) could lead to undesirable immoral behavior. Due to its strong impact on emotional responses, when these are manifest and deliberate, this medium could lead to unwitting manipulation or failure to improve rational thinking, which has led to demands for greater responsibility on the part of producers (Slater and Sanchez-Vives, 2016, p. 34; Bailenson, 2018, p. 206).

This risk is higher in VR experiences whose immediate consequence is triggering emotional contagion or immediate motivation, which we consider the least neutral aspects of empathy. Emotional contagion (personal distress) and empathic concern are frequently biased toward the people closest to us. It is easier for us to share the emotions of our relatives, friends, pets, and the people that we resemble physically. There are kinship and racial variables that also have an influence on the emotional mirroring or triggering process, which is probably a psychological consequence of evolution (de Waal, 2012; Zaki, 2014, p. 1610). In fact, this automatic disposition is present in other animals, especially mammals, for which emotional contagion plays an essential role in parental caring and nurturing (Decety and Cowell, 2014)^12^. Thus, the altruistic motivation triggered by emotional mirroring is often considered a low-level, basic or rudimentary form of empathy. It has also been suggested that these types of empathy are not genuinely significant to morality (see Prinz, 2011a,b, Masto, 2015, p. 76, Bloom, 2016).

In addition to the parochial tendency to empathize more easily with in-group members, empathic concern elicited from emotional mirroring does not necessarily give reasons to act, but mainly *emotions to act*. Therefore, an attempt to enhance human morality by stressing the emotional dimension of empathy would prove unsuccessful and incomplete. By no means are we stating that the examples that we have previously mentioned in this section are ethically dangerous or unworthy. To be fair, in addition to the compelling narratives, most of the activists' VR proposals are founded on accurate descriptions of social reality. What we are claiming is that caution and paused reflection should be exercised even more in the cases in which emotions are the protagonists of the VR experiences. In the Further Ethical Aspects: in Defense of a Reason-Guided Empathy, we will explain in greater detail a defense of rational control over the emotional engagements triggered by empathic processes. Moreover, these previous examples do not necessarily intend to improve empathic abilities themselves, but rather to provoke social changes through the impact that VR technologies might have on empathic responses. This does not take away from their merit, but does make them weaker candidates in the virtual training methods for empathy enhancement.

## Virtual Reality Embodied Perspective-Taking

The world's leading labs that are studying the relationship between VR, empathy and prosocial motivations have been particularly interested in the use of this technology to change participants' perspective through virtual embodiment. There is a growing body of scientific literature providing experimental studies on the so-called “Virtual Reality Perspective-Taking” in the articles of Herrera et al. (2018) and van Loon et al. (2018), which would be similar to the kind of cognitive empathy that Davis called perspective-taking. Here, we will label them “Virtual Reality Embodied Perspective-Taking” (VREPT) in order to add to the relevance of the embodied aspect, which we consider essential to these experiences^13^. In our opinion, this is the most promising form of VR empathy enhancement, both in terms of general efficacy and in terms of ethical permissibility, provided that certain precautions are taken and which we will discuss later. The potential of VREPT for the reduction of some biases and discriminatory attitudes, along with the increase of prosocial and altruistic motivation, is empirically well-supported. However, beyond the consequences and promising results, we believe that it remains controversial if empathy in general, and cognitive empathy in particular, are the causal psychological mechanisms behind those results. In fact, at the end of this section we will present a study of Bedder et al. (2019) that challenges this widespread assumption.

As we have pointed out in the Introduction section, VR offers the possibility of inhabiting another virtual person by means of body transfers into digital avatars. VREPTs can foster empathic abilities, allowing people not only to metaphorically walk a mile in another person's virtual shoes, but also to literally *embody the virtual representation of the specific social target in whom they wish to increase empathy*. This is very important because embodied social cognition theories signal that our bodily depictions, which are fundamental to our social identities, also play a decisive role in the VR empathic processes of merging our self-representations with others (Maister et al., 2015, p. 6; Parsons, 2015, p. 13; and see Bedder et al., 2019). It is easier to empathize with people (targets of the empathic process) who have an analogous body identity (similar representation of the personal body) to the subject that is empathizing. VREPTs therefore boast the undeniable virtue of allowing for the embodiment of virtual out-group avatars with whom we differ in our social and bodily identities. The range of VREPT studies includes experiences such as embodying other skin tones, genders, ages, members of disabled groups, people in situations of extreme social exclusion, and even other species.

The results of these studies are worthy of mention^14^. Seinfeld et al. (2018), for instance, embodied male offenders in female avatars to experience a household scene of virtual verbal abuse and intimidation by a male character with whom they interacted. Prior to the virtual experience, offenders had shown a poor ability to recognize emotions in facial expressions (especially fear in female faces) in comparison to other males in the experiment control condition. After this vivid experience from the perspective of a female victim, domestic abusers reduced their tendency to misclassify facial expressions (which is an empathic deficiency), thus improving their emotion recognition skills, which may prove important to fostering the efficacy of rehabilitation programs.

Moreover, having people embody avatars of very different ages from their own has found, on the one hand, that mothers who had been transferred into a virtual 4-year-old child increased their levels of empathy for them, and even more so if they had been scolded by their virtual mother (Hamilton-Giachritsis et al., 2018). On the other hand, having people embody a virtual elderly person was more effective than a traditional perspective-taking task for reducing ageism in contexts where the intergroup threat was not direct (Oh et al., 2016).

VREPT has also been used to empathize with members of disabled groups. On the one hand, Ahn et al. (2013) found that embodiment in red-green colorblind avatars leads to more helping behavior toward the members of this collective in the real world. Participants invested twice as much time helping them compared to those who had only imagined the experience of being colorblind. On the other hand, another experiment on disability simulation by Chowdhury et al. (2019)—intending to strengthen empathy and positive attitudes toward disabled people—found that the embodied experience via a virtual avatar sitting in a wheelchair led participants to score lower on the Implicit Association Test, consequently exhibiting fewer negative associations toward people with disabilities. The positive change in bias was, moreover, significantly greater under VR HMD conditions than in non-immersive desktop conditions^15^.

Furthermore, Herrera et al. (2018) conducted a pioneer longitudinal study on empathy and VR whose objective was to embody participants in homeless people. It found that over the course of 8 weeks VREPT was more effective in eliciting positive attitudes toward homeless citizens (even signing petitions for help initiatives) than other less immersive perspective-taking or traditional exercises. However, despite the long-lasting prosocial behavior elicited by that VREPT, and even though Herrera et al. (2018) labeled it “long-term empathy,” they also found that the empathic feeling toward the homeless was observed at similar rates under traditional perspective-taking and virtual embodiment conditions.

Racial embodiment has also been one of the most prominent lines of this research. VREPT of light-skinned participants in dark-skinned avatars diminished their implicit racial bias (Peck et al., 2013), with the effect lasting at least 1 week after only one exposure (Banakou et al., 2016). In addition, Hasler et al. (2017) showed that racial embodiment can also help to curb in-group bias in mimicry. They embodied female Caucasian participants in a black virtual body, showing increased mimicry of a black virtual counterpart. This reversed in-group mimicry favoritism was expressed regardless of the participant's level of implicit racial bias and occurred depending on their virtual race, not their actual racial identity. In other words, “[w]hen embodied in a Black virtual body, White participants treat Black as their novel in-group and Whites become their novel out-group” (Hasler et al., 2017, p. 1). This study is interesting with regard to low level empathy because behavior mimicry is related to rudimentary forms of empathic mirroring. It may also be another fruitful starting point for future research on racial embodiment and empathy—although probably not the most interesting with regard to ethics for the reasons already explained at the end of the previous section. In addition to this evidence, VREPT in another race avatar can also lead to negative outcomes. In the first full-body racial embodiment experiment, Groom et al. (2009) reported that Caucasian participants who had embodied dark-skinned avatars showed greater implicit racial bias outside the virtual environment. The racial embodiment was carried out during a job interview—an unpleasant and competitive scenario often associated with racial biases and discriminatory attitudes. This hostile social setting could explain the negative results. According to Hasler et al. (2017, p. 11), the fact that the social interaction involved in the study of Groom et al. (2009) did not occur in a neutral situation explains in some way why implicit racial biases increased after the virtual experiences. More specifically, Bedder et al. (2019, p. 7–8) propose that magnitude and plasticity of implicit bias toward out-groups are modulated by self-esteem due to the novel positive or negative associations generated during the virtual embodiment. In the hostile setting of a job interview, self-esteem is threatened and cognitive associations with the embodied avatar are likely to be negative. This produces a reactive self-identification with characteristics represented by the virtual avatar, such as race, consequently leading to an increase in implicit racial biases after the experience (Bedder et al., 2019, p. 7–8). That said, the moderating effects of the context in VREPT experiences merits further exploration to understand how embodiment in VR might be leveraged to reduce implicit biases and increase empathy. This could also be useful for determining which situations should be avoided in the design of those virtual experiences due to ethical reasons involving potential undesired effects, an issue we will address in the following section.

In addition, Ahn et al. (2016) embodied people in non-human avatars such as virtual cattle^16^ and virtual coral (in the latter case experiencing an acceleration of the ongoing ocean acidification) to promote engagement with environmental problems and foster interconnection with nature. Another notable experiment, while not exactly pursuing the study of perspective-taking, had participants embody human “superheroes” (humanoid avatars with the superpower of flight) to research possible links to the promotion of prosocial behavior. The results showed that the helping behavior of these participants increased in the real world after the experience (Rosenberg et al., 2013).

One of the baseline ideas underlying these experiments is that empathy (especially in its cognitive dimension) is the opposite of a “fixed quality” that is unchangeable (Bailenson, 2018, p. 81–82). Empathic perspective-taking is dependent on individual capabilities that differ from person to person (Ahn et al., 2013, p. 11), but is “like a muscle which one can work to increase its strength” (van Loon et al., 2018, p. 2). This empathy “muscle” can be trained with perspective-taking exercises. Perspective-taking is the psychological process of imagining the world from another person's point of view (Oh et al., 2016, p. 399; Bailenson, 2018, p. 82). It is a “deliberate, effortful activity, and not one that typically occurs automatically” (Davis et al., 1996, p. 719). Empathy is therefore an ability that can also be cultivated (Read, 2019) and directed at will (Persson and Savulescu, 2018, p. 186 and 190). As we have noted, VREPTs are a particularly promising activity to develop perspective-taking capabilities by inhabiting other virtual avatars, this being “more experientially vivid” than just imagining another person's viewpoint (Gehlbach et al., 2015, p. 524). Nevertheless, it should be acknowledged that VR is not the only form of perspective-taking training.

There are various types of traditional perspective-taking techniques for fostering empathy: mental simulations, thought experiments, role-playing exercises, narrative constructions, commenting graphic stories or printed vignettes, certain video games, etc. The core strategy of all of them is to try to imagine another person's perspective. However, these tasks have some shortcomings that should be addressed. First, traditional perspective-taking exercises are an effortful activity and are often cognitively taxing (Davis et al., 1996; Ahn et al., 2013, p. 9; Oh et al., 2016, p. 399), which may seem exhausting to some people. Second, this cognitive effort might create a “motivational hurdle” in many individuals, leading many to avoid attempting to take on the viewpoint of others (Bailenson, 2018, p. 83). Zaki (2014) explained this motivational reluctance to empathize by virtue of three phenomena that the agent wants to avoid: the suffering that would be experienced by identifying with the pain of others; the material costs that helping would entail (in terms of charitable donations, for example), as well as the consequent feeling of guilt if it does not prove helpful; and the detrimental effects on the individual in scenarios of hostile negotiation or inter-group competition. Third, some individuals possess traits or find themselves in personal situations that make it difficult for them to put themselves in the place of others. This may be either because they do not have the necessary skills to do so, or because they lack the information necessary to imagine themselves having experiences not common for them (Ahn et al., 2016, p. 15). On the other hand, there is also a dependency on the background of participants and their knowledge regarding their targets. This is problematic because there is sometimes a need for additional information due to the lack of direct experience that is fundamental to the task; e.g., in the case of imagining a disability such as being red-green colorblind (Ahn et al., 2016, p. 4). Other times, there is a risk of reproducing some biases, erroneous information, or stereotypes about the social target. This is why researchers often prefer to engage participants in a “mediated perspective-taking task,” providing important and accurate information about the targets, leading the exercise appropriately, and not “relying solely on the user's imagination” (Herrera et al., 2018, p. 3).

A clear advantage of VREPT is that it overcomes those previous drawbacks of traditional perspective-taking activities. Since VR is an experience machine, in VREPT users do not have to imagine because they need only concentrate on their actual experience (Banakou et al., 2016, p. 8; Seinfeld et al., 2018, p. 7). Therefore, acting or just being in an IVE is less cognitively demanding than a traditional (mediated or not) perspective-taking task. Moreover, VREPTs are vivid experiences with rich sensorial nuances that provide a greater “degree of realism” (Ahn et al., 2013, p. 10) and “perspectival fidelity” (Ramirez and LaBarge, 2018). Also, this plurality of stimuli makes it more difficult to “escape” from a VREPT than from other perspective-taking exercises in which one is simply thinking from the point of view of another person (Oh et al., 2016, p. 400). From an embodied account to social cognition, this is also important in relation to an accurate understanding of empathy because it relies on the interplay between the performance of one's embodied mind and a specific environment (Herrera et al., 2018, p. 4). For instance, imagining what it would be like to spend time in a maximum-security prison cell from the comfort of one's living room is not the same as undergoing a first-person virtual experience locked behind bars in solitary confinement—even without leaving your living room^17^. Most likely, the process of empathizing with the inmate would be stronger in the second case. Last but not least, VREPTs have a substantial advantage in terms of avoiding “stereotypes and false comforting narratives,” given that the “mental model of perspective of the empathic subject can be created in great detail in VR” (Bailenson, 2018, p. 83).

Nevertheless, caution should be exercised with the results of the VREPTs outlined above and with future undertakings. There is a need for more empirical support to conclude that VREPTs are usually better ways of promoting empathy for specific others than other traditional mediums. In that sense, further VREPT studies should provide evidence including, specifically, empathy-measuring scales—such as the IRI of Davis (1983)—and also comparisons of the added value of virtual embodiment compared to traditional perspective-taking tasks—measuring both characteristics as in the study of Herrera et al. (2018). Furthermore, new studies should address the role that interactivity plays in the enhancement of empathy in VREPTs, that is, whether having agency in IVEs significantly contributes to perspective-taking engagement (Herrera et al., 2018, p. 32). There is also a noteworthy absence of longitudinal studies, a lack of diversity in the sample of some of the research and little knowledge about the medium and long-term effect of the interventions^18^. As Oh and Bailenson (2017, p. 13) point out: “[s]ample populations in academic research typically consist of young, upper-class, college-educated individuals, which makes it difficult to predict the generalizability of in-lab studies. […] future VR studies should address the long-term outcomes of virtual interventions.” Finally, there are certain factors that still need to be further explored. For instance, the novelty factor of VR is something that should be researched thoroughly. The impact of VR might decrease in relation to the level of familiarization with the technology, affecting first-time users, and participants who have more experience with the medium in different ways (Oh et al., 2016, p. 407; Herrera et al., 2018, p. 30–32; Archer and Finger, 2018). Bailenson (2018, p. 44) recalls the urban legend of the early projections of the Lumière Brothers' *The Arrival of a Train at La Ciotat Station*, a 50-second black-and-white short documentary, in which the audience supposedly shouted and ran out when the train on the screen started approaching them, precisely to contemplate the importance of the novelty factor in every new technological advance.

A final observation must be taken into account with regard to these experiments. It remains controversial whether the changes produced by these simulated illusions of body ownership are primarily due to empathy-related phenomena. In fact, in a recent paper by Bedder et al. (2019) it was suggested that at least implicit bias modifications through virtual embodiment might be better explained by a mechanistic account of *bodily resonance*, that is, empathy (in any of its subtypes) would not necessarily be the causal psychological process changing some implicit attitudes. Conversely, bodily resonance might constitute a better explanatory phenomenon. Bodily resonance is the “comparison between cognitive representations of our own self-image and that of the other” (Bedder et al., 2019, p. 1). Self-image representations encode personal features (particularly physical and bodily ones such as gender, skin tone, hair color, etc., but also more abstract characteristics such as group memberships) in neural networks. The cognitive mechanistic account of bodily resonance states that “during subsequent perception of another agent, total output from the self-image network is proportional to the degree of overlap between that agent's features and the encoded self-image” (Bedder et al., 2019, p. 2). Accordingly, bodily resonance could explain not only the existence of implicit attitudes but also their modification in previous VREPT experiences. Virtual embodiment shows that our self-image representations are highly plastic and that they can influence social cognition by modifying implicit biases after the VR experience.

The contribution of the latter study is noteworthy because it indicates that there may be positive (and negative) consequences of VREPT experiences that are not necessarily caused by empathy-related phenomena. Bedder's et al. proposal goes in the same line of other cognition-based explanations—such as the proteus effect of Yee and Bailenson (2007) and the predictive coding models of Maister et al. (2015), Tsakiris (2017), and Farmer and Maister (2017)—which challenge the notion that empathy is the principle underlying mechanism of the effects of virtual embodiment on out-group attitudes and behavior changing. One thing that seems crucial is that perspective-taking through virtual embodiment creates a novel association between oneself and the other. Furthermore, it is precisely the positive or negative self-association with the other, and not necessarily empathy itself, that largely explains the increase or decrease in implicit attitudes, and changes in behavior outside the laboratory [see Farmer and Maister (2017), p. 337, 340–343]. Indeed, mitigating implicit racial biases and strengthening empathy toward people of other races are not the same thing. Nevertheless, having more implicit negative associations is a hurdle to taking on the perspective of others, to feeling what they feel, and to eliciting the subsequent altruistic motivations. Moreover, according to Tsakiris (2017), there are still many unresolved theoretical questions in the domain of empathy and embodiment in the self-other overlapping gap—and in how they affect higher level social processes—in part because direct empirical evidence has only recently begun to be generated. In the face of these theoretical frameworks, and the mixed evidence presented by the previous VREPT experiments, the role of cognitive empathy should not be over-interpreted, but also should not be completely neglected. Further research should be conducted to improve our understanding of the underlying mechanisms that cause different behavioral effects both during and after virtual embodiment.

In summary, we have seen that there is an interdisciplinary body of literature in the area of VR that researches the link between virtual embodiment and different empathic processes, some of which are related to the cognitive ability of perspective-taking. In this respect, it could be said that VREPTs are a *humbly promising* form of empathy enhancement: “humbly” because the research line covering them is quite recent, but “promising” because we already have some empirical evidence of their potential and our knowledge about them is likely to continue growing^19^. However, there is a significant absence of consideration of the ethical aspects that arise from the aim of fostering empathy in these projects. We will approach them from a perspective of moral philosophy in the next section.

## Further Ethical Aspects: in Defense of a Reason-Guided Empathy

Empathy does *not* equate to moral behavior. So, why should we believe that empathy enhancement is often ethically desirable? To answer this question, there are several points that must be addressed. In this last section, we will first set out the moral development and ethical framework to defend the notion that empathy has an important role in human morality, showing that it is also conditionally necessary from an ethical point of view. This will not prevent us from later highlighting its shortcomings. We will address the moral implications of empathy in general, and of VREPTs in particular, and will show some of the most recurrent criticisms of the moral relevance of empathy. These drawbacks must be taken seriously. We will thus propose three ethical requirements for a reason-guided empathy in order to counterbalance these shortcomings. Then, we will point out two advantages which make empathy an ability that very often is morally desirable. Finally, provided that the above requirements are met, we will consider the possibility that VREPTs may be an ethically acceptable technology to improve empathy.

The ordinary and pre-theoretical concept of empathy denotes that empathy is something positive or even desirable (Battaly, 2011). The general folk view is that empathy is necessary because by allowing us to take on the perspective of others and/or to share the feelings of others we are more likely to behave morally, for example, by helping those most in need. However, for some specialized critics, if what is at stake is helping others, this can be achieved without having to evoke empathy. It would suffice to have firm determination to do what reason tells us is correct, and reason will dictate that we all have, for instance, the same right not to suffer. Therefore, to be moral and to attend to others does not require any emotional state or special knowledge about others (McGeer, 2007; Maibom, 2009; Goldie, 2011; Harris, 2013).

Nevertheless, as many philosophers have historically maintained, knowing what and why something is right is no guarantee of being motivated to bring our moral judgments into action. The weakness of will is persistent in human moral psychology. Sometimes we need emotions to move us to do what we are advised to do by reason. Likewise, at other times, as in the case of violent impulses, it is better to be free of the emotions that prevent us from following a reasonable course of action.

In the face of this, there are rationalist authors who have recently argued, in the debate on moral enhancement, that morality cannot be a matter of having the proper emotions. Influencing individuals through biotechnological interventions to do the right thing by relying on better emotions means converting morality into precisely what it ought not to be: a domain where decisions are automatic and lack a deliberative process (Harris, 2010, 2013, p. 172). We agree that morality cannot be reduced to only having the right emotions but we disagree with the premise that, on the contrary, the only thing that matters in morality is reason. Psychology and neuroscience show that moral decision-making is highly complex: cognitive and deliberative aspects are important, but intuition and emotions also play a pivotal role. Actually, the areas involving feelings in our brain are activated when we make moral judgments (Crockett et al., 2010).

A proper criticism of the ethical need for empathy could therefore only come from someone who does not question the relevance of emotions. This is the criticism raised, for instance, by Prinz (2011a, b). This author maintains that morality is essentially based on a strong feeling of disapproval toward certain behaviors that harm others which are generated by identification with the victim or fear of punishment. He argues that to disapprove of such behaviors we need only realize that they are harmful to someone. We agree with Prinz that this is the usual way we act and that, therefore, in most cases, we do not judge an act as wrong because at that moment we empathize with the victim, but rather because we simply disapprove of it. However, this does not invalidate the claim that empathy is a crucial element to adopting this feeling of disapproval.

For the development of moral sense, we need to imagine how others will react to the rules with which we intend to govern our own conduct. To do so, the mere emotional imitation Prinz refers to must be completed and transformed through an understanding of the other's state of mind, especially when it does not correspond to simple emotions. These cognitive abilities related to empathy normally develop in children between 4 and 5 years old, leading them to become progressively involved in cooperative and altruistic activities and to perceive antisocial and aggressive behavior as negative (Batson, 1991; Eisenberg, 2000; Hoffman, 2000; Decety and Meyer, 2008).

The importance of these empathic abilities becomes more noticeable when we look at the cases where they are absent, for instance, in psychopaths. In this situation, the process of conferring specific gravity to the moment of breaking moral norms does not take place. Normal infants, through imitation and emotional contagion, associate behavior that makes others cry with feeling bad. But over time, this mere contagion is complemented with an ability to imagine the reactions of others, which will lead them to relate the suffering of others to morality and, in this way, to recognize the greater prescriptive force of moral rules in comparison with conventional ones (Blair et al., 1995)^20^.

By virtue of all this, we believe that empathy is necessary for moral development in general ethical terms. However, this does not negate the fact that empathy can sometimes be counterproductive and that it often requires some correction. Next, we will observe these ethical disadvantages of empathy and the extent to which VREPT could overcome them and therefore turn empathy training into a desirable conditioned moral enhancement.

The first concern regarding the ethical significance of empathy has already been pointed out at the end of the Virtual Reality as an “Empathy Machine”. Empathy in general, and more particularly the ability to vicariously feel the emotions of others, is biased. The isomorphic response to other people's affective states is conditioned by factors such as social identity or group membership; in other words, we pay more attention to the emotions of people with whom we share familiarity, affiliation, or similarity (Bertrand et al., 2018). While this parochial tendency, a consequence of our evolutionary moral psychology, could prove alarming, it should not entail a rejection of fostering empathy across the board. Moreover, as we have seen, VREPTs offer the possibility of embodying avatars with whom people differ in their social identity, such as gender, skin tone, age, economic class, group membership, biological species, etc^21^. If sharing the emotions of people with other social identities or with whom we do not have regular contact is indeed harder, VREPTs could be used to limit this propensity. In the event it is proven that the cognitive empathic changes are the underlying mechanism of the positive behavioral effects of virtual embodiments described above, VR could offer experiences to empathize with out-group people and other social targets.

Therefore, the first limitation of empathy could be mitigated with a *requirement of target specificity*. This requirement entails that a subject should be assisted in the effort to empathize with a specific target with whom he or she finds it more difficult to empathize when there are good moral reasons to do so. Assuming the VREPT meets this criterion, when one embodies an avatar of another skin color, empathy is not fostered only with respect to that particular individual, but with respect to all the individuals with whom the avatar shares the same skin color and to whom we ascribe the same social group. Accordingly, VREPTs might become a helpful technological tool to reduce sexism, racism, ageism, ableism, speciesism, and other forms of discrimination.

In those cases in which VREPTs are used to foster empathy for specific social targets, an ethical consideration that should be stressed in this regard is the moral responsibility of representation in virtual reality (Brey, 1999, p. 10–13); that is, the responsibility of designers and creators of the experiences to avoid biases and unjustified idealization regarding avatars. It should be remembered that although avatars are digital beings, in these cases they can be considered representatives of real groups. As such, it would be very useful if, prior to the design of these VR empathy enhancement devices, studies were conducted on marginalized groups in which members of these groups actively collaborated and presented their experiences.

Another important implication of this requirement for designers is the acknowledgment of the difficulty of reducing the social and individual complexity of the target in whom we wish to raise empathy. An adequate empathic attitude toward marginalized groups requires recognition that such marginality can be experienced differently by members of the same group. This occurs by virtue of the existence of different personalities and contexts or because one also belongs to another marginalized group. Failure to realize this could lead to a very simple, rigid and stereotyped knowledge of the reality of these groups that could even jeopardize the achievement of highly efficient altruistic behavior. Therefore, it is very important that designers configure devices based on the experiences of different individuals in each group so that the user perceives the plurality of the group and the particular features of its members.

A second precaution that must be considered from an ethical viewpoint is that empathy could lead to morally incorrect behavior. Moral behavior can be incorrect due to specific actions or omissions. Sometimes, in truly serious situations, a personal distress can saturate, paralyze, and even lead to inaction when the morally correct thing to do is to act (Masto, 2015, p. 78–79). Other times, empathic concern can induce an altruistic action that could be considered morally wrong if it is partial and unfair (Batson et al., 2015, p. 17). Some experiments in which the participants were asked to decide on the distribution of resources affecting the well-being of others have shown that those who were induced to feel empathy tended to act in violation of the principles of justice more than those who were not induced to do so, distributing the resources preferentially to the individual toward whom they felt empathy (Batson et al., 1995). It is therefore important to note that prosocial dispositions like empathy are not always morally desirable. This is because of the complex, nuanced, and contextual nature of morality. There are certain types of actions that are right in some situations and wrong in others. Hence, empathizing in some specific situations could be morally desirable, but in others it could lead to immoral consequences. For instance, it would be morally inappropriate to promote a judge's empathy for the accused who she is going to sentence, as this could lead her to unfairly favor the accused.

This second limitation of empathy must also be taken into account when preparing new VREPT experiences. If empathy is not always desirable, there are both virtual and real situations in which the saliency of empathy should not be encouraged. We therefore need a second criterion, that is, the *requirement of context dependency*. This requirement establishes that empathy should be especially strengthened in situations in which empathy is morally important to make proper decisions in order to do the right thing.

The increase of an ethically acceptable empathy does not always occur under the same conditions. Different situational characteristics, social contexts and group, or individual characteristics could give rise to different results when attempting to increase empathy. For instance, experimental studies show that this contextual dependence occurs when oxytocin is used to increase empathy. Empathy increases considerably in contexts of interaction with relatives, but little in situations of competition (Shamay-Tsoory et al., 2009), uncertainty (Declerck et al., 2010), institutional inefficiency (Zak, 2008) and interaction with strangers (De Dreu et al., 2010). Likewise, the use of oxytocin produces differing levels of empathy depending on the personality of the individual to whom it is administered. In particular, it would be less effective for those who were previously less empathic (Abu-Akel et al., 2015) or were less willing to show empathy (Rodrigues et al., 2009; Barraza, 2010); for children who received little attention from their parents (Carter, 2003); for violent people (DeWall et al., 2013); or simply for men rather than women (Hurlemann et al., 2010). Presumably, many of these contextual limitations will also be present when using VREPT instead of oxytocin to enhance empathy. Moreover, the previously discussed experiment by Groom et al. (2009)—in which racial bias was increased when Caucasian participants were embodied in a dark-skinned avatar in a job interview—has shown that non-neutral and competitive contexts may have the opposite undesired effects. If VREPTs aim to have positive results with regard to empathic abilities, prosocial behavior and constructive identification with out-groups, counterproductive virtual situations should be avoided (Farmer and Maister, 2017, p. 343). This second requirement therefore requires that both the use and design of these devices take these possible contextual limitations into account.

A third downside that can be attributed to empathy is that it is neither necessary nor sufficient in many ethical challenges. First, there are many moral problems in which empathizing is not necessary to reach solutions. Most of our current pressing problems are global, such as the environmental crisis that we face today, which is so broad and abstract that it makes it very difficult to feel any empathic concern for the Earth as a target, “even though personalizing metaphors like “Mother Earth” may move us in that direction” (Batson et al., 2015, p. 16–7). In a case like this, it is dubious that empathy would be morally necessary (if not feasible) to address the environmental crisis in a more appropriate way. On the other hand, there are cases in which empathy could prove helpful, or even necessary, but in which it is not sufficient—either for proper decision-making or to envisage the accurate causes and solutions of morally serious problems. For instance, severe poverty is a persistent moral injustice in which empathy could be useful to raise awareness, but that depends on social structures beyond individual initiatives. One could feel empathy for *particular* poor people (when we encounter homeless people on the street, even if we avoid their gaze, or an advertisement of an NGO showing impoverished living conditions in a distant continent)^22^, which could encourage increasing our helping behavior toward them. However, combating poverty depends more on our societal commitments than on individual willingness. Pursuing responsibility for the unfair distribution of resources, excessive wealth accumulation, or the legal and social advantages of rich people is more important to reducing poverty than empathizing with the poor. Moreover, even if the causes were clearly and broadly agreed upon, the solutions should be implemented in political arenas, primarily at a national and supranational level.

This third limitation of empathy should serve as a wake-up call for not placing too much expectation on empathy in general and on VREPTs in particular. In addition, it shows us that we need a third requirement for cases in which greater empathy is necessary, but is by no means sufficient. This third criterion is that of the *requirement of complementarity*. This requirement stipulates that even in cases where promoting empathy is necessary to be a competent moral agent, we should not rely on *it alone* in order to do the right thing. Consequently, VREPTs should not serve as isolated interventions or as substitutes for other initiatives. For instance, if VREPT becomes an effective technology in rehabilitation programs for domestic abusers, these experiences should not be a replacement for other therapies, but rather another complement to the program. Group or individual therapies led by professional psychologists to talk about gender-based violence, sexism, emotional management, and impulse control should not be excluded based on the use of this technology. In fact, Seinfeld's et al. (2018) study was conducted in a therapeutic context in which offenders also attended a weekly therapy group as part of a domestic violence rehabilitation program. In addition, if we agree that more empathy is necessary to, for instance, reduce racial aversion, an issue likely related to brain activity^23^, VREPTs might assist in lessening implicit racial biases, as shown in Peck et al. (2013) and Banakou et al. (2016). However, the most fundamental causes of racism are not biological, but rather social discourses that promote discrimination against people from whom we differ physically, culturally and genealogically. Therefore, while reducing implicit racial biases via technology would be ethically favorable, racism should first and foremost be combated by providing good reasons, including empirical knowledge and ethical values^24^.

These three ethical criteria (the requirements of target-specificity, context-dependency, and complementarity), which aim to counterbalance some of the alleged shortcomings of empathy, constitute the backbone of a *reason-guided empathy*. A reason-guided empathy is, precisely, a concept that could subsume these three requirements. Persson and Savulescu (2018) proposed the concept of *reflective empathy* to refer to the moral significance of an empathy supervised by reason. They argued that if empathy is “harshly disciplined” by factors such as reason it can play an appropriate role in morality, which may be considered ethically important (Persson and Savulescu, 2018, p. 183). Our reason-guided empathy is consequently established along the same lines of reflective empathy. We agree that empathy should be severely disciplined by reason, just as a jockey has to control the horse^25^. After all, human beings have empathy-regulating mechanisms and strategies that are morally desirable (Ray and Castillo, 2019; Read, 2019). This kind of empathy can be ethically advantageous in at least two ways.

The first considerable advantage of a reason-guided empathy is that it can boost the *motivation* to behave morally. Not coincidentally, it has been pointed out that empathy can play an essential role in moral motivation (Masto, 2015; Persson and Savulescu, 2018; Read, 2019). Moral emotions and moral motivation are closely related. Empathy exercises and VREPTs can lead to emotional identifications that can be fundamental to promoting moral impetus^26^. Taking actions in order to try to do the right thing is something that can be elicited by the major dimensions of empathy, namely, emotional contagion, empathic concern, and perspective-taking. Empathic concern, in particular, is the basis for the empathy-altruism hypothesis, which postulates that empathic concerns are related to altruistic motivations due to other-oriented emotional responses triggered by the suffering of beings whose welfare we value (Batson, 2009; Batson et al., 2015). Empathy is therefore sometimes an essential tool to appreciate the welfare of others, perceive negative situations and emotions (such as pain, sadness, distress, sorrow, affliction, etc.) and move us to alleviate such suffering.

It is therefore evident that the effective moral motivation that arises from VREPTs might sometimes be elicited by negative emotions caused by the embodied first-person perception of bad situations. But this also has serious risks. Kross and Ayduk (2017) have attempted to demonstrate that the best strategy for overcoming depression or anxiety derived from negative memories is to face them with emotional self-distance and consider them from a third-person perspective. Negative memories are not positively overcome by distraction or by trying to rationally understand them by remembering them in the first-person. If these authors are correct, it is possible that anyone who experiences the negative feelings of others in order to understand what they are feeling in the first person, through VREPT, could end up experiencing distress or even traumatic effects^27^. The fact that the virtual embodiment is not a true memory of something experienced previously, and that the VR user is consistently aware that their experience does not correspond to a real situation, could reduce the possible adverse effects compared to the subjects of the Kross and Ayduk experiments. However, the mere possibility that the mental well-being of users could potentially be negatively affected in some way forces us to take precautions in the use of these mechanisms as empathy enhancers. These should only be used for this purpose when the expected benefits outweigh the harm caused and when the intensity or duration of the experiences are modulated so as to avoid possible serious damage to users' mental health^28^.

The second upside is that empathy can be a heuristic source of morally relevant information. The *epistemological functions* of empathy are important in human morality (Coplan, 2011; Oxley, 2011; Masto, 2015). Other-oriented empathy is often a reliable (albeit imperfect) source of interpersonal data that might be valuable to accomplish an “experiential understanding” of other beings, that is, “it provides an observer with knowledge of another person's thoughts, feelings, and behavior” (Coplan, 2011, p. 17). Empathy therefore might be used to gather intelligible information about others' minds, states and emotions, thus providing knowledge and understanding and making salient other people's perspectives (Oxley, 2011, p. 33–58). If our personal decisions and behaviors might have an impact on other targets, empathy often proves necessary (though not sufficient) to anticipate their perspective and, consequently, helps in the agent's moral judgment of the right thing to do (Masto, 2015). Generally speaking, empathic abilities in the epistemological domain are useful in informing us of the state of other beings that are morally salient. In fact, this is not an extraordinary characteristic of our moral life, but rather an extremely ordinary operation in our everyday moral considerations (Masto, 2015, p. 87–91). This is because worrying about the welfare or discomfort of others is a fundamental trait of morality that can be elicited through empathy. In the end, *empathy is also a source of reason* that have a place in our moral deliberations.

## Conclusion

Virtual Reality is a technology that offers several applications in different domains of human life, such as education, health, research, leisure time, communication, tourism, activism, journalism, sports, art, etc. The importance of considering the ethical aspects of VR is therefore even greater in light of the increasingly widespread use of this technology (Madary and Metzinger, 2016; Slater and Sanchez-Vives, 2016; Oh and Bailenson, 2017; Bailenson, 2018; Slater et al., 2020). In this article, we have addressed numerous ways to improve empathy-related phenomena through VR experiences. Nonetheless, empathy is not exempt from morally problematic considerations. It can be biased, it can lead to morally incorrect acts or omissions, and it sometimes does not serve us in our moral dilemmas. Nevertheless, it is possible to enhance our conception, ability, and practice of empathy through the guidance of reason. Boosting empathy through technological means can sometimes be ethically desirable. VREPTs can become morally strong candidates if they appropriately specify their social targets, if the context in which empathy needs to be developed is justified and if they are conceived as a necessary complement to other capabilities for doing the right thing. Better moral motivation and reinforcement of moral epistemology are clear advantages that can be attained. Although empathy should not have the last word in decision-making processes, it can help to make more informed decisions and, subsequently, to bring these decisions to action in a motivated way, which we undoubtedly believe would constitute moral enhancement.

## Data Availability Statement

The raw data supporting the conclusions of this article will be made available by the authors, without undue reservation, to any qualified researcher.

## Author Contributions

JR and FL contributed with the bibliography exploration, content development, and writing of the manuscript. Both authors made substantial, direct and intellectual contributions to the article, and approved it for publication.

## Conflict of Interest

The authors declare that the research was conducted in the absence of any commercial or financial relationships that could be construed as a potential conflict of interest.

## Footnotes

^1^More than two decades ago Brey (1999) warned designers and users how certain VR applications that involved performing immoral actions in the virtual environment (e.g., pedophilia) could encourage poor treatment or real psychological harm to people represented by abused avatars. Other ethical aspects of VR have subsequently been considered, including ethical guidelines for research on this type of technology (Behr et al., 2005); the harm to the individual and society, especially to the vulnerable population, derived from the emotional, cognitive, and behavioral changes of the virtual embodiment; the use of highly persuasive technology to get others to perform immoral actions; and issues related to privacy, data sharing, and the misuse of personal data required for the implementation of this technology (Brey, 2008; Wason, 2014; Madary and Metzinger, 2016). Slater et al. (2020) discuss how the ethical issues mentioned above could be exacerbated by the super-realism produced by the latest developments in VR which will continue to reach even greater levels in the near future.

^2^Some biomedical tools for boosting empathy have also been proposed. For instance, see Lara (2017) for the ethical considerations of promoting empathy through oxytocin in the human enhancement debate. For another approach to the ethical aspects of the biomedical enhancement of empathy, see Ray and Castillo (2019).

^3^The term “Virtual Reality” was coined by Jaron Lanier in 1978 (Bailenson, 2018, p. 174).

^4^This is the biggest difference between Virtual Reality and Augmented Reality: “[i]n contrast to VR, which attempts to completely block out the physical world, AR refers to the overlaying of virtual objects onto the physical world” (Oh and Bailenson, 2017, p. 3).

^5^Another distinction worth bearing in mind in this discussion is between dispositional and situational empathy. Dispositional, or so-called trait empathy, refers to individual stable characteristics that can be self-reported to measure the propensity of a person for empathy. By contrast, situational, or state empathy refers to the variable level of empathy that may be experienced according to a particular situation and context. One of the uncertainties is whether recurrent exposure to VR situations, in which empathy is directly elicited, might result in a significant increase in the dispositional traits of empathy in the medium or long-term. For that discussion see https://virtualrealitypop.com/measuring-empathy-in-virtual-reality-678dd02eac61 (accessed January 11, 2020).

^6^A similar case will be shown in the Virtual Reality Embodied Perspective-Taking, in Seinfeld's et al. (2018) experiment, in which male abusers embodied female avatars to experience an episode of gender-based violence at home. Following the experience, male abusers improved their ability to recognize emotions in women's faces.

^7^Even though there is already a national survey experiment in the United States pointing out the positive results of a written perspective-taking exercise encouraging short-term inclusionary behavior toward Syrian refugees (Adida et al., 2018), there is little empirical research on the effects of VR in relation to refugees. See, for instance, Schutte and Stilinović (2017) and Sundar et al. (2017). However, there are also detractors of these types of projects. Paul Bloom, famous for his book *Against Empathy* (2016), published an opinion piece entitled “It's Ridiculous to Use Virtual Reality to Empathize with Refugees” in *The Atlantic* in 2017: https://www.theatlantic.com/technology/archive/2017/02/virtual-reality-wont-make-you-more-empathetic/515511/ (accessed February 12, 2019).

^8^See https://ianimal360.com/ (accessed February 7, 2019).

^9^See https://vrforimpact.com/ and https://www.oculus.com/vr-for-good/ (accessed July 16, 2019).

^10^An interesting question is why VR has been welcomed so enthusiastically with respect to empathy, unlike other areas of technological media. There are two possible explanations for this. The first is that new media and the rise of the internet have been partially blamed for the decrease in the empathic tendencies of the latest generations. In a prominent cross-temporal meta-analysis conducted on American college students between 1979 and 2009, Konrath et al. (2011) showed a considerable decline on the Empathic Concern and Perspective-taking subscale of these young adults, according to the Multidimensional Interpersonal Reactivity Index (Davis, 1980, 1983) of dispositional empathy scale. That study also indicated that both affective and cognitive empathy had sharply dropped after 2000. Comparing 1979 with 2009, the subscale score had decreased by 48% in Empathic Concern and by 34% in Perspective-taking in American college students (Konrath et al., 2011, p. 186). This decline correlates, on the one hand, with the increased mass consumption of cable television, new internet media, smartphones, and self-promoting social networks, and, on the other, with changes in self-oriented traits such as the increase in psychological scales of individualistic self-esteem and narcissism (Konrath, 2013). Secondly, we believe that there is more confidence in VR than in these other new types of media, as a form of empathy enhancer, precisely because it is believed that VR technology has a greater potential for immersion, embodiment, and emotional impact on the user.

^11^Furthermore, the potential of easily provoking emotional responses is not only a feature of 360° VR videos. Felnhofer et al. (2015) demonstrated how computer-generated virtual scenarios can purposely lead to different affective states (e.g., joy, boredom, anger, or anxiety). They study how the creation of different virtual parks may intentionally attempt to condition mood and agitation by producing diverse levels of psychophysiological arousal.

^12^Empathic concern is likewise a consequence of evolution (Decety and Cowell, 2014; Batson et al., 2015, p. 6). See also de Waal (2012).

^13^Bertrand et al. (2018) also argued that the embodied dimension is crucial for the potential to learn empathy-related abilities in VR. They speak about the use of immersive embodied virtual reality (EVR) for this purpose. Schoeller et al. (2019) also use the EVR acronym.

^14^There was an earlier experiment in which the embodiment aspect was not exploited, but whose objective was to reduce the negative stigma attached to people with schizophrenia through VR. Kalyanaraman et al. (2010) discovered that experiencing the point of view of a person with schizophrenia (through a VR simulation of auditory and visual hallucinations while visiting a grocery store pharmacy), in addition to a thought-listing exercise, induced the highest empathy score of all experimental conditions. It is a meaningful example of the potential for VR users to walk in the shoes of people with severe mental illnesses.

^15^The *Projet RÉVE* (RÉalité Virtuelle et Empathie), supported by the French Ministry of Education and conducted with the help of Be Another Lab, is also attempting to develop the use of VR to raise awareness in teachers about some disabilities in students, such as offering immersive experiences from the perspective of a dyslexic pupil. See https://www.education.gouv.fr/110bislab/cid143142/projet-reve-realite-virtuelle-et-empathie.html (accessed September 26, 2019).

^16^Ramirez and LaBarge (2018, p. 522) has been skeptical about experiments that have virtually embodied participants in very different ways of existence, such as in species that differ considerably from humans like cows. The famous article *What Is it Like to Be a Bat?* by Nagel (1974) could reinforce this skepticism.

^17^To see different VR films that denounce the United States prison system visit https://www.projectempathyvr.com/ (accessed July 16, 2019). See also Bang and Yildirim (2018).

^18^In this respect, the inquiry of Herrera et al. (2018) is a notable exception. This study worked with a large sample of a diverse population, abandoning the typical university campus lab in which most of the participants in the experiments are students at the college, and using a mobile-lab unit. The effects of the VR empathy interventions were also studied over the course of 8 weeks.

^19^Something similar is stated by Louie et al. (2018, p. 750–1) about this “nascent” and “promising” literature.

^20^An objection to this could arise from the case of autistic people who, despite their lack of empathy, can come to respect moral rules. However, empirical studies suggest that the moral norms governing the behavior of autistic people are perceived by them as conventional norms. They explain moral wrongness in terms of the violation of a norm but without relating this to physical or emotional harm to others (De Vignemont and Frith, 2007; Hobson et al., 2009). They have some emotional responses but these cannot be empathic either because they lack the capacity to imagine themselves in the minds of others and because of their persistent self-centered perspective (De Vignemont and Frith, 2007).

^21^We might wonder if the use of VREPTs for empathy enhancement might be susceptible to ethically questionable use. Isn't there a risk that this technology will be developed and eventually used to increase our empathy not toward the discriminated people or victims of aggression, but toward the discriminators or aggressors? Given our previously-mentioned understanding of the important role that empathy plays in our moral development and the fact that this basically consists of associating the strength of moral rules with the protection of all people from suffering and harm, we believe that, in general terms, this potential use of VREPTs to empathize with abusers would be ineffective. Still, some studies suggest that some people, under certain circumstances (such as previous similar experiences as an abuser or gender ties), would be surprisingly willing to empathize with rapists and corrupt people (see Konrath and Grynberg, 2016). Moreover, this technology may have unforeseen effects that are ethically reprehensible and should be anticipated by the designer. Bearing in mind that in the experience of virtually taking the perspective of another, we tend to get carried away by the facts that we are witnessing and not by the causes motivating the facts. This may sometimes prevent the understanding of exceptional, justifiable aggressions or apparent discriminations and therefore lead to a misplaced empathy toward the victim.

^22^According to Bloom (2016), empathy is a spotlight that permits us to focus on certain people here and now, but is shortsighted and innumerate. It thus facilitates the ability to empathize with specific people, but not with the suffering of groups, favoring the one over the many.

^23^Gazzaniga (2005) and Douglas (2008, 2013) mentioned some neuroscientific studies that show a relationship between the amygdala and racial aversion.

^24^In some cases, complementary tasks are not only an ethical requirement, but also necessary in terms of efficacy to reduce negative stereotypes and improve empathy levels. For instance, this was specifically highlighted in the experiment on schizophrenia conducted by Kalyanaraman et al. (2010, p. 441–442) in which the highest score of empathy was achieved with the VR experience plus an extra thought-listing exercise.

^25^It should be deduced from the beginning of this Further Ethical Aspects: in Defense of a Reason-Guided Empathy that we are not defending an extreme rationalist conception of the relationship between empathy and reason. We believe that a normative point of view unconnected to the perspectives and suffering of others can only lead to behaviors close to the cold egocentrism of the psychopath or, in the ethical field, to a simplistic and naïve rationalism. Therefore, we defend the need for an inescapable complementarity between these two factors that ultimately leads us to affirm that “empathy should be severely disciplined by reason” and vice versa.

^26^By no means are we suggesting that emotions are the only source of moral motivation. On the contrary, we believe that proper reasoning can also be morally motivating and that sound arguments might have a strong persuasive power.

^27^This is also important in relation to the motivational outcomes of VREPTs. According to Batson et al. (1997), self-focus perspective-taking (imagining how you would feel in another's situation) and other-focused perspective-taking (imaging how another feels in a situation) have different emotional and motivational consequences. Imagine-other perspective-taking evokes empathy-related altruistic motivation. Imagine-self perspective-taking, on the other hand, not only evokes altruistic motivation but can also cause personal distress with the subsequent egoistic motivation, which inhibits helping behavior. Actually, it has also been said that genuine empathy is the other-oriented form (Coplan, 2011). It is difficult to establish a relationship between that account and VREPT for two reasons. First, because VREPT potentially blurs the distinction between the self-focus and other-focus perspectives. Second, VREPTs are showing an overarching tendency to elicit altruistic motivations even though the overall experience in most of the experiments was not traumatic. Admittedly, more research is needed to predict whether the distress caused by VREPT experiences can lead to egoistic outcomes.

^28^We would like to thank the reviewers for raising this point.

## References

[B1] Abu-Akel A. Palgi S. Klein E. Decety J. Shamay-Tsoory S. (2015). Oxytocin increases empathy to pain when adopting the other–but not the self-perspective. Soc. Neurosci. 10, 7–15. 10.1080/17470919.2014.948637 25103924

[B2] Adida C. L. Lo A. Platas M. L. (2018). Perspective-taking can promote short-term inclusionary behavior toward syrian refugees. Proc. Natl. Acad. Sci. U.S.A. 115, 9521–9526. 10.1073/pnas.1804002115 30181296 PMC6156655

[B3] Ahn S. J. Bostick J. Ogle E. Nowak K. L. McGillicuddy K. T. Bailenson J. N. (2016). Experiencing nature: embodying animals in immersive virtual environments increases inclusion of nature in self and involvement with nature. J. Comput. Mediat. Commun. 21, 399–419. 10.1111/jcc4.12173

[B4] Ahn S. J. Le A. M. Bailenson J. (2013). The effect of embodied experiences on self-other merging, attitude, and helping behaviour. Media Psychol. 16, 7–38. 10.1080/15213269.2012.755877

[B5] Archer D. Finger K. (2018). Walking in Another's Virtual Shoes: Do 360-Degree Video News Stories Generate Empathy in Viewers? New York, NY: Tow Center for Digital Journalism, Columbia University 10.7916/D8669W5C

[B6] Arora G. Pousman B. (2015). Clouds Over Sidra. Available online at: https://www.youtube.com/watch?v=FFnhMX6oR1Q (accessed February 6, 2019).

[B7] Bailenson J. (2018). Experience on Demand. What Virtual Reality Is, How It Works, and What It Can Do. New York, NY; London: W. W. Norton & Company.

[B8] Bailey J. O. Bailenson J. N. Flora J. Armel K. C. Voelker D. Reeves B. (2014). The impact of vivid messages on reducing energy consumption related to hot water use. Environ. Behav 47, 570–592. 10.1177/0013916514551604

[B9] Banakou D. Hanumanthu P. D. Slater M. (2016). Virtual embodiment of white people in a black virtual body leads to a sustained reduction in their implicit racial bias. Front. Hum. Neurosci. 10:601. 10.3389/fnhum.2016.00601 27965555 PMC5126081

[B10] Bang E. Yildirim C. (2018). “Virtually empathetic? Examining the effects of virtual reality storytelling on empathy,” in Virtual, Augmented and Mixed Reality: Interaction, Navigation, Visualization, Embodiment, and Simulation. 10th International Conference, VAMR 2018 Held as Part of HCI International 2018 Las Vegas, NV, USA, 2018 Proceedings, Part I, eds. J. Y. C. Chen and G. Fragomeni (Cham: Springer), 290–298.

[B11] Barraza J. (2010). The Physiology of Empathy: Living Oxytocin to Empathic Responding. (Dissertation), Claremont, CA: Claremont Graduate University, Proquest.

[B12] Batson C. (1991). The Altruism Question: Toward a Social-Psychological Answer. Hillsdale: Lawrence Erlbaum.

[B13] Batson C. D. (2009). “These things called empathy: Eight related but distinct phenomena,” in The Social Neuroscience of Empathy, eds. J. Decety and W. Ickes (Cambridge, MA: MIT Press), 3–16.

[B14] Batson C. D. Early S. Salvarani G. (1997). Perspective taking: imagining how another feels versus imagining how you would feel. Pers. Soc. Psychol. Bull. 23, 751–758. 10.1177/014616729723700816140345

[B15] Batson C. D. Klein T. R. Highberger L. Shaw L. L. (1995), Immorality from empathy-induced altruism: when compassion justice conflict. J. Pers. Soc. Psychol. 68, 1042–1054. 10.1037/0022-3514.68.6.1042

[B16] Batson C. D. Lishner D. A. Stocks E. L. (2015). “The empathy-altruism hypothesis,” in The Oxford Handbook of Prosocial Behavior, eds. D. A. Schroeder and W. G. Graziano (Oxford: Oxford University Press), 259–281.

[B17] Batson C. D. Oleson K. (1991). “Current status of the empathy-altruism hypothesis,” in Prosocial Behavior, ed M. Clark (Thousand Oaks, CA: Sage)

[B18] Battaly H. D. (2011). “Is empathy a virtue?,” *in Empathy: Philosophical and Psychological Perspectives*, eds P. Goldie and A. Coplan (New York, NY: Oxford University Press), 277–301.

[B19] Bedder R. L. Bush D. Banakou D. Peck T. Slater M. Burgess N. (2019). A mechanistic account on bodily resonance and implicit bias. Cognition 184, 1–10. 10.1016/j.cognition.2018.11.010 30553934 PMC6346146

[B20] Behr K. M. Nosper A. Klimmt C. Hartmann T. (2005). Some practical considerations of ethical issues in vr research. Presence 14, 668–679. 10.1162/105474605775196535

[B21] Bertrand P. Guegan J. Robieux L. MacCall C. A. Zenasni F. (2018). Learning empathy through virtual reality: multiple strategies for training empathy-related abilities using body ownership illusions in embodied virtual reality. Front. Robot. AI 5:26 10.3389/frobt.2018.00026 PMC7805971 33500913

[B22] Blair R. J. R. Jones L. Clark F. Smith M. (1995). Is the psychopath ‘morally insane'? Pers. Individ. Dif. 19, 741–752. 10.1016/0191-8869(95)00087-M

[B23] Bloom P. (2016). Against Empathy. London: Bodley Head.

[B24] Bollmer G. (2017). Empathy machines. Media Int. Aust. 165, 63–76. 10.1177/1329878X17726794

[B25] Brey P. (1999). The ethics of representation and action in virtual reality. Ethics Inf. Technol. 1, 5–14. 10.1023/A:1010069907461

[B26] Brey P. (2008). “Virtual Reality and Computer Simulation,” in The Handbook of Information and Computer Ethics, eds. K. Himma, and H. T. Tavani (Hoboken, NJ: Wiley), 361–384. 10.1002/9780470281819.ch15

[B27] Carter C. (2003). Developmental consequences of oxytocin. Physiol. Behav. 79, 383–397. 10.1016/S0031-9384(03)00151-3 12954433

[B28] Chartrand T. Barh J. (1999). The chameleon effect: the perception-behavior link and social interaction. J. Pers. Soc. Psychol. 76, 893–910. 10.1037/0022-3514.76.6.893 10402679

[B29] Chowdhury T. I. Ferdous S. M. S. Quarles J. (2019). VR disability simulation reduces implicit bias towards persons with disabilities. IEEE Trans. Vis. Comput. Graph. 10.1109/TVCG.2019.2958332. [Epub ahead of print]. 31825867

[B30] Coplan A. (2011). “Understanding empathy: its features and effects,” in Empathy: Philosophical and Psychological Perspectives, eds. P. Goldie and A. Coplan (New York, NY: Oxford University Press), 3–18.

[B31] Crockett M. J. Clark L. Hauser M. D. Robbins T. W. (2010). Reply to harris and chan: moral judgment is more than rational deliberation. Proc. Natl. Acad. Sci. U.S.A. 107:E184 10.1073/pnas.1015402107 PMC2951447 20876101

[B32] Cruz-Neira C. Sandin D. J. DeFanti T. A. (1993). “Surround-screen projection-based virtual reality: the design and implementation of the CAVE,” in Proceedings of the 20th Annual Conference on Computer Graphics and Interactive Techniques (Anaheim, CA), 135–142.

[B33] Cuff B. M. P. Brown S. J. Taylor L. Howat D. J. (2016). Empathy: a review of the concept. Emot. Rev. 8, 144–153. 10.1177/1754073914558466

[B34] Cummings J. J. Bailenson J. (2016). How immersive is good enough? A meta-analysis of the effect of immersive technology on user presence. Media Psychol. 19, 272–309. 10.1080/15213269.2015.1015740

[B35] Darwall S. (1998). Empathy, sympathy, care. Philos. Stud. 89, 261–282. 10.1023/A:1004289113917

[B36] Davis M. H. (1980). A multidimensional approach to individual differences in empathy. Catal. Select. Doc. Psychol. 10:85.

[B37] Davis M. H. (1983). Measuring individual differences in empathy: evidence for a multidimensional approach. J. Pers. Soc. Psychol. Bull. 9, 223–229. 10.1177/0146167283092005

[B38] Davis M. H. Conklin L. Smith A. Luce C. (1996). Effect of perspective taking on the cognitive representation of persons: a merging of self and other. J. Pers. Soc. Psychol. 70, 713–726. 10.1037/0022-3514.70.4.713 8636894

[B39] De Dreu C. K. Greer L. L. Handgraaf M. J. Shalvi S. Van Kleef G. A. Baas M.. (2010). The neuropeptide oxytocin regulates parochial altruism in intergroup conflict among humans. Science 328, 1408–1411. 10.1126/science.1189047 20538951

[B40] De Vignemont F. Frith U. (2007). “Autism, morality and empathy”, in The Neuroscience of Morality: Emotion, Brain Disorders, and Development, ed W. Sinnot-Armstong (Cambridge: MIT Press).

[B41] de Waal F. B. M. (2012). The antiquity of empathy. Science 336, 874–876. 10.1126/science.1220999 22605767

[B42] Decety J. Cowell J. M. (2014). Friends or foes: is empathy necessary for moral behavior? Pers. Psychol. Sci. 9, 525–537. 10.1177/1745691614545130 25429304 PMC4241340

[B43] Decety J. Meyer M. (2008). From emotion resonance to empathic understanding: a social developmental neuroscience account. Dev. Psychopathol. 20, 1053–1080. 10.1017/S0954579408000503 18838031

[B44] Declerck C. Boone C. Kiyonari T. (2010). Oxytocin and cooperation under conditions of uncertainty: the modulating role of incentives and social information. Horm. Behav. 57, 368–374. 10.1016/j.yhbeh.2010.01.006 20080100

[B45] DeGrazia D. (2014). Moral enhancement, freedom, and what we (should) value in moral behaviour. J. Med. Ethics 40, 361–368. 10.1136/medethics-2012-101157 23355049

[B46] DeWall C. N. Gillath O. Pressman S. D. Black L. L. Bartz J. A. Moskovitz J. (2013). When the love hormone leads to violence: oxytocin increases intimate partner violence inclinations among high trait aggressive people. Soc. Psychol. Personal. Sci. 20, 1–6. 10.1177/1948550613516876

[B47] Douglas T. (2008). Moral enhancement. J. Appl. Philos. 25, 228–245. 10.1111/j.1468-5930.2008.00412.x 19132138 PMC2614680

[B48] Douglas T. (2013). Moral enhancement via direct emotion modulation: a reply to John Harris. Bioethics 27, 160–168. 10.1111/j.1467-8519.2011.01919.x 22092503 PMC3378474

[B49] Eisenberg N. (2000). Emotion, regulation, and moral development. Annu. Rev. Psychol. 51, 665–697. 10.1146/annurev.psych.51.1.665 10751984

[B50] Ekman P. (1992). Facial expressions: new findings, new questions. Psychol. Sci. 3, 34–38. 10.1111/j.1467-9280.1992.tb00253.x

[B51] Ekman P. Davidson R. (1993). Voluntary smiling changes regional brain activity. Psychol. Sci. 4, 42–45. 10.1111/j.1467-9280.1993.tb00576.x

[B52] Farmer H. Maister L. (2017). Putting ourselves in another's skin: using the plasticity of self-perception to enhance empathy and decrease prejudice. Soc. Justice Res. 30, 323–354. 10.1007/s11211-017-0294-1

[B53] Faust H. S. (2008). Should we select for genetic moral enhancement? A thought experiment using the moral kinder (MK+) haplotype. Theor. Med. Bioethics 29, 397–416. 10.1007/s11017-008-9089-6 19132548

[B54] Felnhofer A. Kothgassner O. D. Schmidt M. Heinzle A.-K. Beutl L. Hlavacs H. (2015). Is virtual reality emotionally arousing? Investigating five emotion inducing virtual park scenarios. Int. J. Hum. Comput. Stud. 82, 48–56. 10.1016/j.ijhcs.2015.05.004

[B55] Fisher J. A. (2017). “Empathic actualities: toward a taxonomy of empathy in virtual reality,” in Interactive Storytelling. ICIDS 2017. Lecture Notes in Computer Science, vol. 10690, eds. N. Nunes, I. Oakley, and V. Nisi (Cham: Springer), 233–44.

[B56] Francis K. B. Gummerum M. Ganis G. Howard I. S. Terbeck S. (2018). Virtual Morality in the helping professions: simulated actions and resilience. Br. J. Psychol. 109, 442–465. 10.1111/bjop.12276 29164607

[B57] Gazzaniga M. S. (2005). The Ethical Brain. New York, NY: Dana Press.

[B58] Gehlbach H. Marietta G. King A. M. Karutz C. Bailenson J. Dede C. (2015). Many ways to walk a mile in another's moccasins: type of social perspective taking and its effect on negotiation outcomes. Comput. Hum. Behav. 52, 523–532. 10.1016/j.chb.2014.12.035

[B59] Giubilini A. Savulescu J. (2017). The artificial moral advisor. The ‘ideal observer' meets artificial intelligence. Philos. Technol. 31, 169–188. 10.1007/s13347-017-0285-z 29974033 PMC6004274

[B60] Goldie P. (2011). “Anti-empathy: against empathy as perspective-shifting,” in Empathy: Philosophical and Psychological Perspectives, eds P. Goldie and A. Coplan (New York, NY: Oxford University Press) 302–317.

[B61] Groom V. Bailenson J. Nass C. (2009). The influence of racial embodiment on racial bias in immersive virtual environments. Soc. Infl. 4, 231–248. 10.1080/15534510802643750

[B62] Hamilton-Giachritsis C. Banakou D. Garcia Quiroga M. Giachritsis C. Slater M. (2018). Reducing risk and improving maternal perspective-taking and empathy using virtual embodiment. Sci. Rep. 8:2975. 10.1038/s41598-018-21036-2 29445183 PMC5813089

[B63] Harris J. (2010). Moral enhancement and freedom. Bioethics 25, 102–111. 10.1111/j.1467-8519.2010.01854.x 21133978 PMC3660783

[B64] Harris J. (2013). ‘Ethics is for bad guys!' Putting the ‘moral' into moral enhancement. Bioethics 27, 169–173. 10.1111/j.1467-8519.2011.01946.x 22296666

[B65] Harris J. (2016). How to be Good. The Possibility of Moral Enhancement. Oxford: Oxford University Press.

[B66] Hasler B. S. Spanlang B. Slater M. (2017). Virtual race transformation reverses racial in-group bias. PLoS ONE 12:e0174965. 10.1371/journal.pone.0174965 28437469 PMC5403166

[B67] Heeter C. (1992). Being there: the subjective experience of presence. Presence 1, 262–271. 10.1162/pres.1992.1.2.262

[B68] Herrera F. Bailenson J. Weisz E. Ogle E. Zaki J. (2018). Building long-term empathy: a large scale comparison of traditional and virtual reality perspective-taking. PLoS ONE 13:e0204494. 10.1371/journal.pone.0204494 30332407 PMC6192572

[B69] Hobson J. A. Harris R. García-Pérez R. Hobson R. P. (2009). Anticipatory concern: a study in autism. Dev. Sci. 12, 249–263. 10.1111/j.1467-7687.2008.00762.x 19143798

[B70] Hoffman M. (2000). Empathy and Moral Development: The Implications for Caring and Justice. Cambridge: Cambridge University Press.

[B71] Hurlemann R. Patin A. Onur O. A. Cohen M. X. Baumgartner T. Metzler S.. (2010). Oxytocin enhances amygdala-dependent, socially reinforced and emotional empathy in humans. J. Neurosci. 30, 4999–5007. 10.1523/JNEUROSCI.5538-09.2010 20371820 PMC6632777

[B72] Kalyanaraman S. S. Penn D. L. Ivory J. D. Judge A. (2010). The virtual doppelganger. Effects of a virtual reality simulator on perceptions of schizofrenia. J. Nerv. Mental Dis. 198, 437–443. 10.1097/NMD.0b013e3181e07d66 20531123

[B73] Klincewizc M. (2016). Artificial intelligence as a means to moral enhancement. Stud. Logic Grammar Rhetoric. 48, 171–187. 10.1515/slgr-2016-0061

[B74] Konrath S. (2013). “The empathy paradox: increasing disconnection in the age of increasing connection,” in Handbook of Research on Technoself : Identity in a Technological Society, ed R. Luppicini (Hershey, PA: Information Science Reference). 967–991.

[B75] Konrath S. Grynberg D. (2016). “The positive (and negative) psychology of empathy,” in Psychology and Neurobiology of Empathy, eds D. Watt and J. Pankseep (New York, NY: Nova Science Publishers), 63–107.

[B76] Konrath S. O'Brien E. H. Hsing C. (2011). Changes in dispositional empathy in american college students over time: a meta-analysis. Pers. Soc. Psychol. Rev. 15, 180–198. 10.1177/1088868310377395 20688954

[B77] Kross E. Ayduk O (2017). Chapter two – self-distancing: theory, research, and current directions. Adv. Exp. Soc. Psychol. 55, 81–136. 10.1016/bs.aesp.2016.10.002

[B78] Lara F. (2017). Oxytocin, empathy and human enhancement. Theoria: an international journal for theory. Hist. Foundations Sci. 32, 367–384. 10.1387/theoria.17890

[B79] Lara F. Deckers J. (2020). Artificial intelligence as a socratic assistant for moral enhancement. Neuroethics 13, 275–287. 10.1007/s12152-019-09401-y

[B80] Louie A. K. Coverdale J. H. Balon R. Beresin E. V. Brenner A. M. Guerrero A. P. S.. (2018). Enhancing empathy: a role for virtual reality? Acad. Psychiatry 42, 747–752. 10.1007/s40596-018-0995-2 30324398

[B81] Madary M. Metzinger T. K. (2016). Real virtuality: a code of ethical conduct. Recommendations for good scientific practice and the consumers of VR-technology. Front. Robot. AI. 3 10.3389/frobt.2016.00003

[B82] Maibom H. (2009). Feeling for others: empathy, sympathy, and morality. Inquiry 52, 483–499. 10.1080/00201740903302626

[B83] Maister L. Slater M. Sanchez-Vives M. V. Tsakiris M. (2015). Changing bodies changes minds: owning another body affects social cognition. Trends Cogn. Sci. 19, 6–12. 10.1016/j.tics.2014.11.001 25524273

[B84] Maselli A. Slater M. (2013). The building blocks of the full body ownership illusion. Front. Hum. Neurosci. 7:83. 10.3389/fnhum.2013.00083 23519597 PMC3604638

[B85] Masto M. (2015). Empathy and its role in morality. South. J. Philos. 53, 74–94. 10.1111/sjp.12097

[B86] McGeer V. (2007). “Varieties of moral agency: lessons from autism (and psychopathy),” in The Neuroscience of Morality: Emotion, Brain Disorders, and Development, ed W. Sinnott-Armstrong (Cambridge: MIT Press), 227–257.

[B87] Milk C. (2015). How Virtual Reality Can Create the Ultimate Empathy Machine. TED Talk. Available online at: https://www.ted.com/talks/chris_milk_how_virtual_reality_can_create_the_ultimate_empathy_machine#t-120978 (accessed December 28, 2018).

[B88] Nagel T. (1974). What is it like to be a bat? Philos. Rev. 83, 435–450. 10.2307/218391411759714

[B89] Neumann R. Strack F. (2000). ‘Mood contagion': the automatic transfer of mood between persons. J. Pers. Soc. Psychol. 79, 211–223. 10.1037/0022-3514.79.2.211 10948975

[B90] Oh S. Y. Bailenson J. (2017). “Virtual and augmented reality,” in The International Encyclopedia of Media Effects, eds. P. Rössler, C. A. Hoffner and L. Zoonen (John Wiley & Sons), 1–16.

[B91] Oh S. Y. Bailenson J. Weisz E. Zaki J. (2016). Virtually old: embodied perspective taking and reduction of ageism under threat. Comput. Hum. Behav. 60, 398–410. 10.1016/j.chb.2016.02.007

[B92] Oxley J. C. (2011). The Moral Dimensions of Empathy. Limits and Application in Ethical Theory and Practice. New York, NY: Palgrave Macmillan.

[B93] Parsons T. D. (2015). Virtual reality for enhanced ecological validity and experimental control in the clinical, affective and social neurosciences. Front. Hum. Neurosci. 9:660. 10.3389/fnhum.2015.00660 26696869 PMC4675850

[B94] Peck T. C. Seinfeld S. Aglioti S. M. Slater M. (2013). Putting yourself in the skin of a black avatar reduces implicit racial bias. Conscious. Cogn. 22, 779–787. 10.1016/j.concog.2013.04.016 23727712

[B95] Persson I. Savulescu J. (2008). The perils of cognitive enhancement and the urgent imperative to enhance the moral character of humanity. J. Appl. Philos. 25, 162–177. 10.1111/j.1468-5930.2008.00410.x

[B96] Persson I. Savulescu J. (2012). Unfit for the Future. The Need for Moral Enhancement. Oxford: Oxford University Press.

[B97] Persson I. Savulescu J. (2018). The moral importance of reflective empathy. Neuroethics 11, 183–193. 10.1007/s12152-017-9350-7 29881473 PMC5978797

[B98] Prinz J. (2011a). “Is empathy necessary for morality?,” in Empathy: Philosophical and psychological perspectives, eds P. Goldie and A. Coplan (New York: Oxford University Press), 211–229.

[B99] Prinz J. (2011b). Against empathy. South. J. Philos. 49, 214–233. 10.1111/j.2041-6962.2011.00069.x

[B100] Ramirez E. J. (2017). Empathy and the limits of thought experiments. Metaphilosophy 48, 504–526. 10.1111/meta.12249

[B101] Ramirez E. J. LaBarge S. (2018). Real moral problems in the use of virtual reality. Ethics Inf. Technol. 20, 249–263. 10.1007/s10676-018-9473-5

[B102] Ray K. Castillo L. G. (2019). Moral bioenhancement, social biases, and the regulation of empathy. Topoi 38, 125–133. 10.1007/s11245-017-9468-6

[B103] Read H. (2019). A typology of empathy and its moral forms. Philos. Compass 14:e12623 10.1111/phc3.12623

[B104] Rodrigues S. Saslow L. García N. John O. Keltner D. (2009). Oxytocin receptor genetic variation relates to empathy and stress reactivity in humans. Proc. Natl. Acad. Sci. U.S.A. 706, 21437–21441. 10.1073/pnas.0909579106 19934046 PMC2795557

[B105] Rosenberg R. S. Baughman S. L. Bailenson J. (2013). Virtual superheroes: using superpowers in virtual reality to encourage prosocial behaviour. PLoS ONE 8:e55003. 10.1371/journal.pone.0055003 23383029 PMC3559425

[B106] Sánchez-Vives M. V. Slater M. (2005). From presence to consciousness through virtual reality. Nat. Rev. Neurosci. 6, 332–339. 10.1038/nrn1651 15803164

[B107] Savulescu J. Maslen H. (2015). “Moral enhancement and artificial intelligence: moral AI?” in Beyond artificial intelligence. The Disappearing Human-Machine Divide, eds. J. Romportl, E. Zackova and J. Kelemen (New York, NY: Springer), 79–95.

[B108] Schoeller F. Bertrand P. Gerry L. J. Jain A. Horowitz A. H. Zenasni F. (2019). Combining virtual reality and biofeedback to foster empathic abilities in humans. Front. Psychol. 9:2741. 10.3389/fpsyg.2018.02741 30804868 PMC6370744

[B109] Schutte N. S. Stilinović E. J. (2017). Facilitating empathy through virtual reality. Motiv. Emot. 41, 708–712. 10.1007/s11031-017-9641-7

[B110] Seinfeld S. Arroyo-Palacios J. Iruretagoyena G. Hortensius R. Zapata L. E. Borland D.. (2018). Offenders became the victim in virtual reality: impact of changing perspective in domestic violence. Sci. Rep. 8:2692. 10.1038/s41598-018-19987-7 29426819 PMC5807352

[B111] Seinfeld S. Feuchtner T. Maselli A. Müller J. (2020). User representations in human-computer interaction. Hum. Comput. Inter. 10.1080/07370024.2020.1724790. [Epub ahead of print]

[B112] Shamay-Tsoory S. Fischer M. Dvash J. Harari H. Perach-Bloom N. Levkovitz Y. (2009). Intranasal administration of oxytocin increases envy and schadenfreude (gloating). Biol. Psychiatry 66, 864–870. 10.1016/j.biopsych.2009.06.009 19640508

[B113] Shriram K. Oh S. Y. Bailenson J. (2017). “Virtual reality and prosocial behavior,” in Social Signal Processing, eds. J. K. Burgoon, N. Magnenat-Thalmann, M. Pantic and A. Vinciarelli (Cambridge: Cambridge University Press), 304–316.

[B114] Simmons A. (2014). In Defense of the Moral Significance of Empathy. Ethical Theory Moral Prac. 17, 97–111. 10.1007/s10677-013-9417-4

[B115] Slater M. (2009). Place illusion and plausibility can lead to realistic behaviour in immersive virtual environments. Philos. Trans. R. Soc. B Biol. Sci. 364, 3549–3557. 10.1098/rstb.2009.0138 19884149 PMC2781884

[B116] Slater M. Antley A. Davison A. Swapp D. Guger C. Barker C.. (2006). A virtual reprise of the stanley milgram obedience experiments. PLoS ONE 1:e39. 10.1371/journal.pone.0000039 17183667 PMC1762398

[B117] Slater M. Gonzalez-Liencres C. Haggard P. Vinkers C. Gregory-Clarke R. Jelley S. (2020). The ethics of realism in virtual and augmented reality. Front. Virt. Real. 1:1 10.3389/frvir.2020.00001

[B118] Slater M. Sanchez-Vives M. V. (2016). Enhancing our lives with immersive virtual reality. Front. Robot. AI 3:74 10.3389/frobt.2016.00074

[B119] Slater M. Spanlang B. Sanchez-Vives M. V. Blanke O. (2010). First person experience of body transfer in virtual reality. PLoS ONE 5:e10564. 10.1371/journal.pone.0010564 20485681 PMC2868878

[B120] Sundar S. S. Kang J. Oprean D. (2017). Being there in the midst of the story: how immersive journalism affects our perceptions and cognitions. Cyberpsychol. Behav. Soc. Netw. 20, 672–682. 10.1089/cyber.2017.0271 29125787

[B121] Tsakiris M. (2017). The multisensory basis of the self: from body to identity to others. Q. J. Exp. Psychol. 70, 597–609. 10.1080/17470218.2016.1181768 27100132 PMC5214748

[B122] van Loon A. Bailenson J. Zaki J. Bostick J. Willer R. (2018). Virtual reality perspective-taking increases cognitive empathy for specific others. PLoS ONE 13:e0202442. 10.1371/journal.pone.0202442 30161144 PMC6116942

[B123] Wason B. (2014). Augmented Reality Law, Privacy, and Ethics: Law, Society, and Emerging AR Technologies. Waltham, MA: Syngress.

[B124] Weinel J. Cunningham S. Pickles J. (2018). “Deep subjectivity and empathy in virtual reality: a case study on the autism TMI virtual reality experience,” in New Directions in Third Wave Human-Computer Interaction: Volume 1-Technologies, eds M. Filimowicz and V. Tzankova (Cham: Springer), 183–203.

[B125] Whitby B. (2011). “On Computable Morality. An Examination of Machines as Moral Advisors,” in Machine Ethics, eds. M. Anderson and S. L. Anderson, S. L. (Cambridge: Cambridge University Press), 138–150.

[B126] Won A. S. Bailenson J. Lanier J. (2015b). “Homuncular flexibility: the human ability to inhabit nonhuman avatars,” in Emerging Trends in the Social and Behavioral Science: An Interdisciplinary, Searchable, and Linkable Resources, eds. R. A. Scott, S. M. Kosslyn and M. Buchmann (Hoboken, NJ: John Wiley & Sons), 1–16.

[B127] Won A. S. Bailenson J. Lee J. Lanier J. (2015a). Homuncular flexibility in virtual reality. J. Comput. Mediat. Commun. 20, 241–259. 10.1111/jcc4.12107

[B128] Yee N. Bailenson J. (2007). The proteus effect: the effect of transformed self-representation on behavior. Hum. Commun. Res. 33, 271–290. 10.1111/j.1468-2958.2007.00299.x

[B129] Zahn-Waxler C. Robinson J. (1995). “Empathy and guilt: early origins of feelings of responsibility”, in Self-Conscious Emotions, eds. J. Tangney and K. Fischer, (New York, NY: Guilford), 143–173.

[B130] Zak P. (2008). Moral Markets: The Critical Role of Values in the Economy. Princeton: Princeton University Press.

[B131] Zaki J. (2014). Empathy: a motivated account. Psychol. Bull. 140, 1608–1647. 10.1037/a0037679 25347133

